# Smart magnetic resonance imaging-based theranostics for cancer

**DOI:** 10.7150/thno.57004

**Published:** 2021-08-07

**Authors:** Beatriz Brito, Thomas W. Price, Juan Gallo, Manuel Bañobre-López, Graeme J. Stasiuk

**Affiliations:** 1 Department of Imaging Chemistry and Biology, School of Biomedical Engineering and Imaging Sciences, King's College London, Strand, London, UK, SE1 7EH.; 2 School of Life Sciences, Faculty of Health Sciences, University of Hull, Cottingham Road, Hull, UK, HU6 7RX.; 3 Advanced Magnetic Theranostic Nanostructures Lab, International Iberian Nanotechnology Laboratory, Av. Mestre José Veiga, 4715-330 Braga.

**Keywords:** theranostics, responsive, smart, magnetic resonance imaging, cancer, contrast agents, therapy, nanoparticles, small molecules

## Abstract

Smart theranostics are dynamic platforms that integrate multiple functions, including at least imaging, therapy, and responsiveness, in a single agent. This review showcases a variety of responsive theranostic agents developed specifically for magnetic resonance imaging (MRI), due to the privileged position this non-invasive, non-ionising imaging modality continues to hold within the clinical imaging field. Different MRI smart theranostic designs have been devised in the search for more efficient cancer therapy, and improved diagnostic efficiency, through the increase of the local concentration of therapeutic effectors and MRI signal intensity in pathological tissues. This review explores novel small-molecule and nanosized MRI theranostic agents for cancer that exhibit responsiveness to endogenous (change in pH, redox environment, or enzymes) or exogenous (temperature, ultrasound, or light) stimuli. The challenges and obstacles in the design and *in vivo* application of responsive theranostics are also discussed to guide future research in this interdisciplinary field towards more controllable, efficient, and diagnostically relevant smart theranostics agents.

## Introduction

Theranostics is an exciting new field of medicine that refers to the combination of diagnosis and therapy in a single molecule or entity 1. Theranostic agents provide a way to image, characterize, and monitor molecular events while simultaneously delivering a specific therapy 1, 2. These structures have the potential to improve the efficacy of treatment of several disorders, including different types of cancer 3, 4, neurodegenerative disorders 5-7, and cardiovascular diseases 8, 9, thus delivering better personalised medical care 3, 5, 8, 9. Theranostic agents usually combine one or more therapeutic modalities, such as chemotherapy, radiotherapy (RT), photodynamic therapy (PDT), photothermal therapy (PTT), magnetic hyperthermia (MH), immunotherapy or chemodynamic therapy, with different imaging techniques, including magnetic resonance imaging (MRI), positron emission tomography (PET), single photon emission computed tomography (SPECT), ultrasound (US), photoacoustic imaging (PA) or fluorescence imaging (FL) 1, 10, 11.

**Figure 1:**
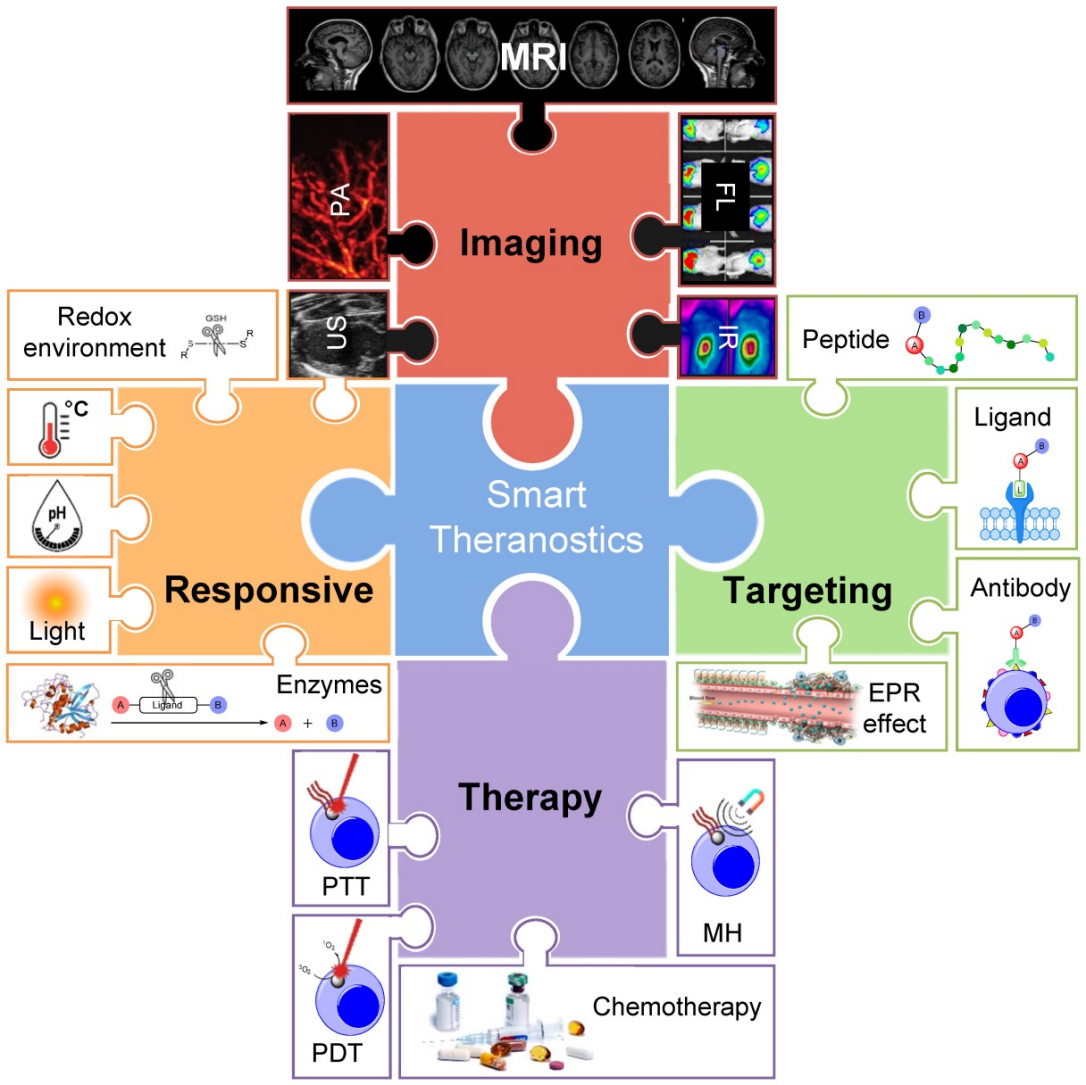
Illustration representing some of the key components of a smart theranostic platform. Smart theranostic systems need to combine contrast agents for distinct imaging modalities, a targeting strategy, therapy effectors and responsive moieties. Adapted with permission from 42-46, copyright 2020 Moran and Thomson, 2018 Stylogiannis et al, 2020 Picillo et al, 2013 Cheng et al, 2016 Tang et al.

The combination of diagnosis/monitoring and therapy can offer synergistic advantages in comparison to standard imaging or treatment alone. Well-designed theranostic agents can facilitate the diagnosis and treatment of diseases at an early stage, provide valuable feedback of drug distribution to target sites and enable monitoring of the response to therapy 12, 13. The efficiency of these theranostic agents is highly dependent on their therapeutic effect, thus several therapeutic modalities are often combined into a single agent 14-16. The efficacy of theranostic agents' imaging capabilities greatly depends on their ability to accumulate at the target site and induce a local enhanced contrast effect. Both the therapeutic and imaging indexes can be improved by designing targeted probes, usually through combination with targeting antibodies 17-19, peptides 20-22, or aptamers 23-25, and/or by designing “smart” responsive theranostic platforms 26-33. The latter type of theranostic agents undergo structural or physicochemical alterations in response to a predefined stimulus (Figure 1) 26, 34.

Smart or responsive theranostics are platforms in which the therapeutic agent is ideally released/activated only after reaching the target tissue, in response to a certain stimulus. These stimuli can be caused by pathological conditions at the target sites compared to healthy tissues, such as changes in the pH, redox environment, overexpression of enzymes, or hypoxic conditions 26, 34. Alternatively, smart theranostics can respond to locally induced external stimuli, such as irradiation with light 35-40 or exposure to magnetic fields 41.

There have been several promising reports of smart theranostic agents for cancer using PET 47, FL 48, 49, and US 50 as the chosen imaging modality 51. However, this review will focus on MRI-based responsive theranostics that are specific for cancer, as MRI is one of the preferred clinical imaging techniques in the diagnosis of this pathology 52-55. This review will also feature relevant dual modal imaging theranostic agents featuring MRI as one of the imaging technologies. We will first review examples of different strategies that have been exploited to design targeted and/or smart contrast agents. Then, we will show how some of those strategies, as well as other novel approaches, have been employed to design a wide range of smart MRI theranostics, which will be divided according to the stimuli that induces drug release and/or magnetic resonance (MR) signal switch. Additionally, we will comment on the obstacles that have so far hindered the clinical implementation of these agents, which include specific targeting limitations, the low sensitivity of the imaging component and reduced biocompatibility. Finally, novel strategies that could be used to overcome these drawbacks will be presented.

## Magnetic Resonance Imaging

MRI is a non-invasive, non-ionising, tomographic imaging modality that is particularly useful to detect and characterize soft tissue pathologies 56, 57. MRI provides three-dimensional (3D) images with high spatial resolution, high penetration and high contrast 56. This makes MRI one of the first choices for clinical diagnosis and monitoring of a wide range of disorders, including cancer, heart disease and neurological or musculoskeletal disorders, among others 52, 58, 59.

The term “MRI” usually refers to proton (^1^H) MRI, which makes use of nuclear properties of water protons present in the body, such as the nuclear spin, to generate images 55. There are other MRI techniques that are sensitive to heteroatoms like fluorine (^19^F), carbon (^13^C) or sodium (^23^Na) 60. ^19^F-MRI in particular is being explored as a promising imaging tool 61.

### Basic principles of MR

The basic principles of heteroatom MRI are similar to those of ^1^H MRI, which are detailed in several reviews and book chapters 55, 56, 62-64. Briefly, after exposure to an external magnetic field (*B_0_*), the magnetic moments of the nuclear spins of water protons align along the longitudinal plane. Then, a radiofrequency (RF) pulse is used to energise the system and to “flip” the magnetic moment of proton nuclei towards the transversal plane. Once the RF pulse is turned off, the transverse magnetisation decays over time through a process known as relaxation, which generates the signals that can be reconstructed into 3D images 56, 63. The relaxation process follows two distinct, independent, and simultaneous pathways: *T_1_* and *T_2_* relaxation 52, 56. Different tissues in the body have distinct *T_1_* and *T_2_* relaxation times and this difference is exploited to create contrast in MR images 52. However, given the high abundance of water molecules in the body, the signal-to-background ratio of MRI is very low. This contributes to the intrinsic low sensitivity of MRI 52.

### MRI contrast agents

Contrast agents (CAs) have been widely used to enhance contrast between tissues and help overcome the sensitivity issue, thus facilitating the diagnosis of different pathologies 65, 66. MRI CAs usually have electronic configurations with unpaired electrons that change local nuclei relaxation time and generate extra contrast in MRI. These MRI CAs can be divided into two categories, depending on their effect on the relaxation of the signal: *T_1_* agents or *T_2_* agents 56, 67.

*T_1_* agents shorten predominantly the *T_1_* relaxation time by withdrawing excess energy from the spin-lattice. This fast recovery of longitudinal magnetisation leads to the production of a strong MR signal and translates into brighter images. *T_1_* agents are usually paramagnetic complexes of the lanthanide ion gadolinium (Gd^3+^), or of the transition metal ion manganese (Mn^2+^) 68-84. There have also been reports of nanoparticles acting as *T_1_* enhancers, including Gd^3+^-based nanoparticles, extremely small iron oxide nanoparticles (ESIONs) and Mn-based nanoparticles 85-90.

Other CAs exert a higher effect on *T_2_* than *T_1_* relaxation, therefore they are referred to as *T_2_* agents 52. These agents shorten the *T_2_* relaxation time, leading to a faster dephasing, which is caused by an increase in spin-spin interactions between the water protons and the CA. The shortening of *T_2_* leads to the production of a weaker MR signal and, consequently, to the procurement of darker images. These agents are typically superparamagnetic iron oxide nanoparticles (SPION) 91-93.

The efficacy of *T_1_* and *T_2_* contrast agents can be determined by their relaxivity (*r_1_* and *r_2_*, respectively), which describes the extent to which a CA can affect the relaxation rate of water and is measured in per millimolar per second (mM^-1^ s^-1^) 52, 59.

Even though MRI CAs significantly increase the contrast between tissues and are a valuable diagnostic tool, the inherent low sensitivity of MRI is still a problem 52. In order to overcome this limitation, there has been an increasing interest in the design of CAs with high relaxivity and/or that specifically accumulate in the tissue of interest 52, 83, 84. As an alternative, the design of switchable MRI CAs has also been explored to increase the signal-to-background ratio at the target site 52, 77, 86, 94-98.

## Smart Theranostics for Cancer

**Figure 2:**
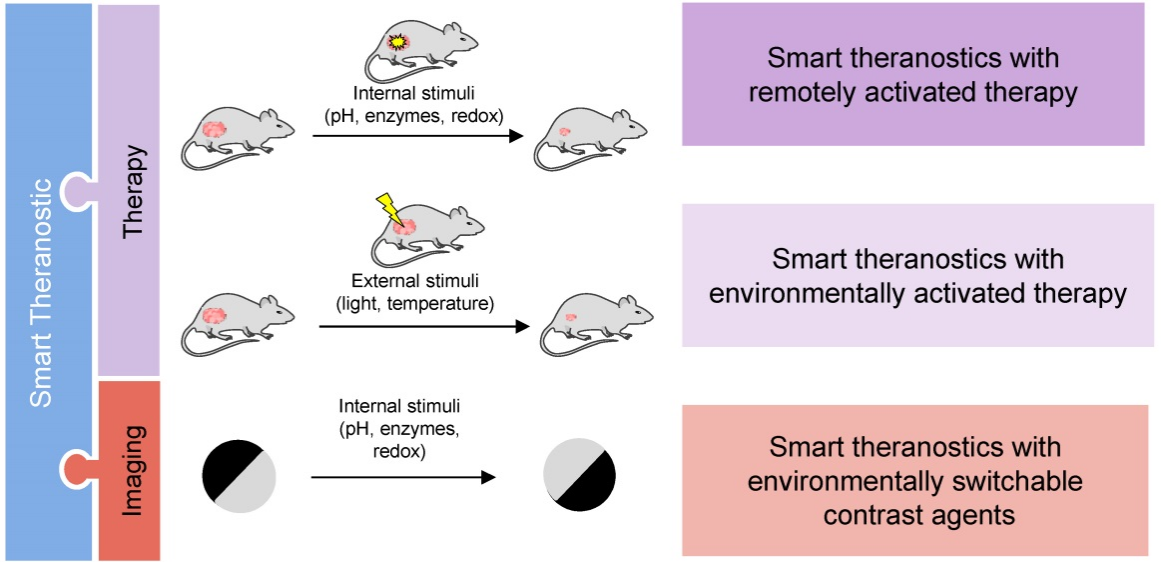
Schematic of the different classifications given to smart theranostic agents based on the effect of the stimulus on their imaging and therapeutic moieties.

Smart theranostics are usually made of at least two components, the therapeutic and the imaging agent moieties, where at least one of them is responsive to environmental conditions or to external stimuli. Smart theranostics can be classified based on the nature of the stimulus (e.g. pH responsive smart theranostics), which is how this review approaches them. However, it is also important to differentiate smart theranostics based on the component that is changed upon exposure to said stimulus (Figure 2).

Both the imaging and therapy components can be altered by a trigger, sometimes simultaneously. Theranostics with responsive therapeutic effect can be divided into two categories: a) smart theranostics with environmentally activated therapy, in which the drug release and/or therapeutic efficacy is triggered by an endogenous signal, e.g. altered enzymatic expression profiles, high glutathione (GSH) concentrations, acidic conditions, or aberrant hypoxia in tumours 26, 34, 99-104; or b) smart theranostics with remotely activated therapy, in which the therapeutic response is triggered by an external signal, e.g. light for PDT agents.

Smart theranostic platforms can also include switchable contrast enhancing components in which the signal enhancement of the imaging agent moiety responds to environmental triggers by switching from off/ON, ON/off or on/ON as the drug is released. In the first type of smart CAs, the relaxation enhancement changes from an “off” state, where contrast enhancement is negligible, to an “ON” state with a significantly higher contrast enhancement. CAs can also undergo stimuli-dependent changes that significantly decrease their relaxivity and contrast enhancement; they are described as ON/off smart CAs. The on/ON contrast switch is common for small-molecule based CAs and describes the change from a state with a relevant, albeit low, relaxation enhancement (“on” state) to one with significantly higher relaxation enhancement (“ON” state). These platforms are classified in this review as smart theranostics with environmentally switchable contrast agents. This classification of theranostics allows for a more efficient monitoring of drug release and response to treatment, better understanding of drug distribution and better chance to personalise treatment, which, in turn, can positively impact patient prognosis.

A smart theranostic agent can be made of a combination of these types. For example, Liu *et al.* designed nanowreaths for PTT and MR that contained imaging agents that were released in response to GSH to impact image contrast, as will be explained later 105. As such, this is an example of a smart theranostic platform with remotely activated therapy and environmentally switchable CAs.

In terms of the characteristics that constitute an ideal smart theranostic agent for cancer, these platforms should aspire to satisfy the requirements for both optimal contrast agents for MRI and excellent therapeutic effectors for the targeted disease, while also being responsive to a specific trigger 106. However, this can be exceedingly difficult to achieve and, as such, the optimised smart theranostic design will most likely result from a compromise of some properties in favour of others. Still, an ideal smart MRI theranostic for cancer should, in broad terms:

- Present good physicochemical stability, biocompatibility and biodegradability;
- Induce strong contrast enhancement;
- Have increased and specific tumour uptake and accumulation;
- Have higher toxicity towards cancer cells versus normal cells;
- Undergo an emphatic change in response to an endogenous or controllable stimulus;
- Have sufficiently long blood circulation time to accumulate at target sites but not so long as to trigger safety concerns.

In this next section, we will explore different theranostic platforms and discuss their advantages and disadvantages.

### pH responsive smart theranostics

The pH of the tumour microenvironment is more acidic than that of normal tissues (pH_tumour_ = 6.4-7.0, pH_healthy_ = 7.2-7.5) 107, 108. This can be exploited to design pH responsive theranostic systems that preferentially release their cargos at lower pH. These platforms usually present at least one component that is ionizable at low pH, and thus induces a physical transformation of nano-based agents that leads to drug release and/or induces a change in the relaxivity of the MR moiety. Other pH-responsive strategies make use of pH-sensitive chemical bonds 109, 110 or acid-degradable nanocomposites to induce drug release and/or relaxation changes 102, 111, 112. Several pH-responsive theranostic agents have been proposed 32, 113-118.

**Figure 3:**
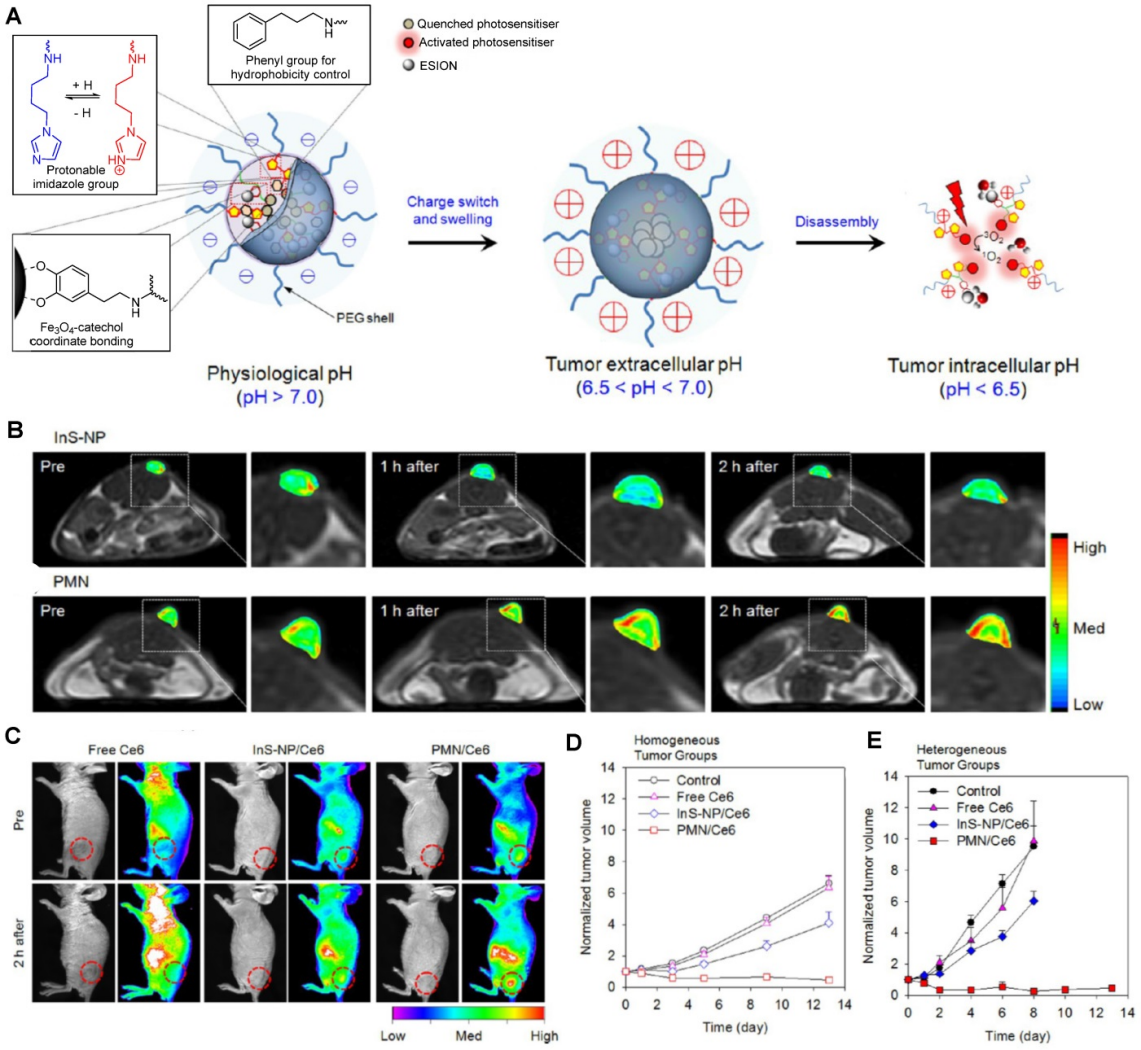
**A.** Schematic illustration of pH-dependent structural transformation of PMNs. The theranostics are latent in the circulation and then the surface charge switches from negative to positive in response to the low pH at the tumour microenvironment, which facilitates tissue permeation and triggers cell internalisation, after which the even more decreased pH induces further disassembly to enhance the MRI contrast and photoactivity of the PMNs. **B.** *In vivo T_1_*-weighted MR images and colour-mapped images of tumour sites before, 1 h after, or 2 h after intravenous injection of PMNs or InS-NPs (2 mg Fe kg^-1^) into nude mice bearing 3-5 mm HCT116 tumours at 1.5 T. **C.** *In vivo* NIR imaging of nude mice bearing HCT116 tumours after intravenous injection of PMNs, InS-NPs or free Ce6 (equivalent to 0.2 mg kg^-1^ Ce6). **D.** Homogeneous HCT116 and **E.** heterogeneous CT26 tumour volumes after treatment with saline (control), free Ce6, InS-NPs and the nanogrenades (PMN/Ce6), followed by irradiation of the tumour regions with a 670 nm laser (250 mW cm^-2^, 20 min) 12 h post injection and 5 days after that. Adapted with permission from 113, copyright 2014 American Chemical Society.

For example, Ling *et al.* synthesised what they called pH-sensitive magnetic nanogrenades (PMNs) that showed pH-sensitive MRI and fluorescence contrast enhancement and could be used for PDT 113. These nanosystems were synthesised by self-assembly of extremely small iron oxide nanoparticles (ESIONs, 3 nm diameter), porphyrin-like photosensitizers for PDT (chlorin e6, Ce6) and contained pH responsive ionizable moieties, polyelectrolytes, on the surface (Figure 3A). These PMNs work in a two-stage pH dependent activation. At physiological pH, the stable nanoparticles are negatively charged. However, when the nanoparticles extravasate to the acidic tumour microenvironment (pH = 6.4-7.0), the polarity of the complex is reversed, due to the ionization of imidazole groups on their surface, which potentiates the increase of electrostatic interactions with anionic cells and subsequent cell internalisation of the complexes into endosomes. Inside endosomes, the pH is lower than 6.5, which triggers complete dissociation of the theranostic platforms.

This is accompanied by an increase in the *r_1_* of PMNs, due to higher protonation of ligands as the pH decreases and consequent increase of water molecules coordinated to Fe^3+^ ions on the surface of ESIONs. The *r_2_/r_1_* ratio similarly decreases during the pH-responsive disassembly process, which contributes to the recovery of *T_1_* MR signal at pH = 5.5 and thus allows for pH sensitive *T_1_* contrast enhancement. The accumulation of PMNs inside tumour cells can be observed in the *T_1_* weighted and NIR images in Figure 3B and C, where a contrast enhancement in both imaging modalities can be seen. This contrast is even perceptible in ultrasmall tumours (3-5 mm) and it is much more notable for the PMNs than for pH-insensitive nanoparticle assemblies (InS-NP). The therapeutic efficiency of these theranostic agents for PDT was also evaluated. Irradiation with a 670 nm laser 12 h after injection with PMN/Ce6 nanosystems led to the destruction of tumour tissue, microvasculature, and fibroblasts in both homogeneous and heterogeneous tumours, unlike irradiation after treatment with pH-insensitive nanoparticle assemblies. This is mainly due to the activation of the self-quenched photosensitisers of the PMN/Ce6 platforms at the low pH of tumour cells or microenvironment. This is an example of responsive nanoplatforms with environmentally (pH) switchable imaging agents and remotely (light) activated therapy, in which pH targeting plays a vital role in improving their imaging properties and therapeutic efficacy.

ESIONs were also used in the design of pH-responsive MRI theranostics that targeted integrin-expressing cancer cells 119. Shen *et al* first synthesised ESIONs by co-precipitation with poly(acrylic acid) (PAA) and studied the effect of the size of the nanoparticles on their relaxivity. The nanoparticles with the best imaging potential (3.6 nm) were then conjugated with a RGD peptide. Poly(ethylene glycol)methyl ether (mPEG) was grafted to the nanoparticles through an acid-labile β-thiopropionate linker, which was followed by conjugation of DOX. This smart design ensures that the integrin ligand is hidden in the mPEG “stealth” layer until the nanoparticles reach the acidic tumour microenvironment, at which point the linker holding the mPEG to the NPs is broken and the exposed RGD peptide binds to α_ν_β_3_-expressing cancer cells. Treatment of U87MG tumour-bearing mice with these DOX@ES-MION3@RGD_2_@mPEG3 constructs showed that the nanoparticles had high MRI imaging efficiency, likely due to high tumour accumulation, and high therapeutic efficacy, since tumour growth was effectively suppressed in all treated mice. This constitutes an example of a biocompatible theranostic with environmentally responsive and targeted drug delivery in which the MR contrast enhancement in tumours is improved by the pH-dependent accumulation on these sites.

Although these iron-based nanoparticles should be more biocompatible than some of the available alternatives (e.g. Gd-based systems), they could still cause toxic effects *in vivo*. The toxicity of these type of nanoparticles is usually dependent on specific characteristics of the nanoparticles, including chemical composition, size and surface to volume ratio, immunogenicity, and pharmacokinetics 120. As such, these factors should be carefully studied in smart theranostic design.

He *et al.* also designed theranostic agents with environmentally (pH) activated therapy by loading gadolinium oxide (Gd_2_O_3_) nanoparticles as MRI agents and doxorubicin (DOX) as a chemotherapy drug inside mesoporous silica nanoparticles (MSNs) modified with polyelectrolytes and folic acid on the surface 115. These nanoplatforms enter cells specifically by folic acid receptor mediated endocytosis and accumulate inside endosomes. The acidic pH of endosomes triggers the disintegration of the polyelectrolytes on the surface of the nanoparticles and leads to the release of DOX, which was confirmed by time-resolved fluorescence microscopy. The MRI contrast is also increased for these targeted systems, due to the accumulation of Gd_2_O_3_ nanoparticles (*r_1_*= 9.14 mM^-1^ s^-1^ at 3.0 T) inside folic acid receptor expressing cells. In these types of Gd^3+^-based nanosystems, as well as in Gd^3+^ complexes, the stability, biosafety and biocompatibility should be ensured, as Gd-based CAs have been linked to nephrogenic systemic fibrosis (NSF) and free Gd^3+^ ions are known to be toxic 121, 122.

**Figure 4:**
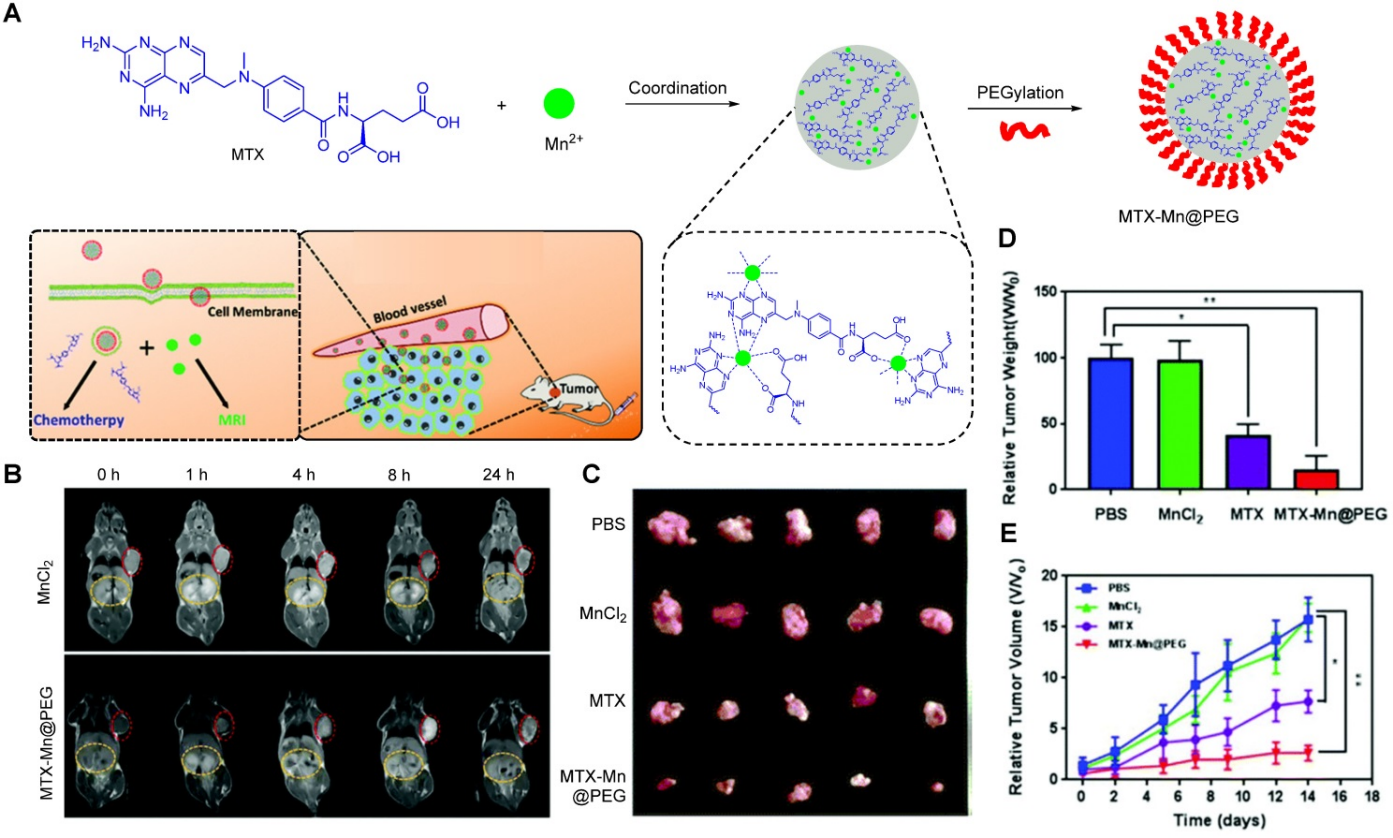
** A** Schematic illustration of the composition of MTX-Mn@PEG nanocapsules and their use as a theranostic nanoplatform for MRI guided chemotherapy. **B** *In vivo T_1_* MR images of MnCl_2_ and MTX-Mn@PEG at different times post injection; red circles indicate the tumour and yellow circles indicate the kidneys of the mice, at 0.5 T. **C** Photographs of tumours excised from mice after 14 days of treatment. **D** Relative tumour weight of each group compared to the PBS group. **E** Relative tumour volumes of mice during the treatment. Adapted with permission from 117, copyright 2013, Royal Society of Chemistry.

Recently, Wu *et al.* fabricated a chelate-free, pH responsive nanoscale coordination polymer with Mn^2+^ ions coordinated to chemotherapy drug methotrexate (MTX) and coated with PEG (MTX-Mn@PEG, Figure 4A) 117. Pharmacokinetic assays in mice showed that these nanoparticles had enhanced circulation half-life, as they lasted up to 4 ± 2 h in blood (in comparison, MnCl_2_ lasted only 0.71 ± 0.15 h). This increase in blood circulation was attributed to the stabilising effect of the PEG coating and means that the particles have more time to accumulate in the tumour site through the enhanced permeability and retention (EPR) effect, which leads to a contrast enhancement in MRI that can last up to 24 h post injection in mice (Figure 4B). The pH dependent drug release mechanism was also investigated. The authors attributed the faster drug release at low pH (48.9% release after 4 h at pH = 5.5 versus 33.2% release after 12 h at pH = 7.4) to the loss of coordination of Mn^2+^ to the chemotherapy drug, following protonation of the carboxylic groups and nitrogen heterocycle of MTX at low pH. This pH dependent mechanism and the accumulation of the MTX-Mn@PEG nanoparticles at the tumour sites contributed to a more efficient tumour regression than treatment with MTX alone, as seen on Figure 4C, D and E.

This is a rather straightforward method to synthesise theranostic nanosystems for cancer therapy that can be adapted to other drugs, metals, or even other imaging modalities. However, the pH that induces drug release (pH = 5.5) is relatively low when compared to the pH of most tumour microenvironments (pH = 6.4-7.0), which limits the responsiveness of the system, as the drug is probably only released following cellular uptake. It is also important to consider the thermodynamic and kinetic stability of the metal-drug complexes, as other paramagnetic metals, such as Gd^3+^, can present serious toxicity problems upon complex dissociation 123-127. Indeed, even free Mn^2+^ can impart neurotoxicity in mice and has a median lethal dose (LD_50_) of 0.3 mmol kg^-1^ in these animals when injected intravenously 70. Even though it is unlikely that this LD_50_ will be reached after administration of 200 µL of MnCl_2_ (0.23 mg mL^-1^) or of the nanotheranostic (1 mg mL^-1^), the dosage of MTX-Mn@PEG nanoparticles still needs to be carefully considered.

**Figure 5:**
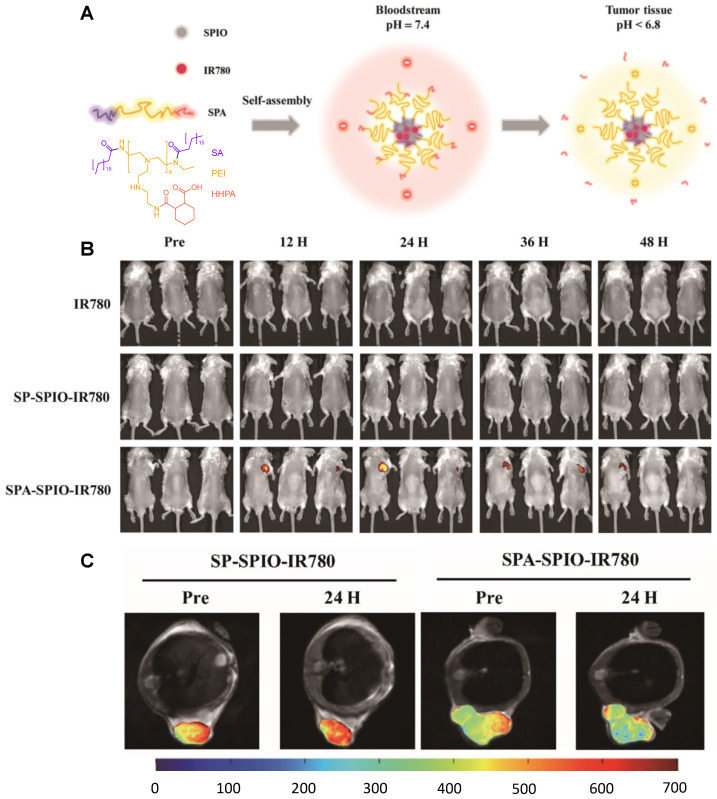
**A** Schematic illustration of the mechanism of the pH-sensitive nanotheranostic system. (SPIO: superparamagnetic iron oxide nanocrystals, SA: stearic acid, PEI: polyethylenimine, HHPA: hexahydrophthalic anhydride, SPA: hexahydrophthalic anhydride modified stearic acid-grafted polyethylenimine. **B** Time-lapse NIR images of mice treated with IR780, SP-SPIO-IR780 or SPA-SPIO-IR780 nanosystems, respectively. **C** MRI (colour mapping) of the tumour site before and 24 hours post injection of SP-SPIO-IR780 and SPA-SPIO-IR780, 3.0 T. Adapted with permission from 116, copyright 2013 Royal Society of Chemistry.

pH-responsive theranostics can also be fashioned for PTT and/or PDT. For example, Wang *et al.* designed a theranostic nanosystem that could be used for MRI/near infrared imaging (NIR)-guided PTT/PDT of triple negative breast cancer 116. This work aimed to develop theranostic platforms for the diagnosis of tumour location and precise phototherapy navigation, as well as for the determination of the therapeutic window of time and prognosis. Their pH-responsive theranostic nanosystem was composed of a hydrophobic core made of SPIONs and IR780 dye, surrounded by a hydrophilic crown (SPA, Figure 5A) of stearic acid, polyethylenimine (PEI) and hexahydrophthalic anhydride (HHPA). The responsiveness of the theranostic is introduced by the SPA matrix, which dissociates in the presence of a faintly acidic environment (pH = 6.5). At this pH, the amido bond between PEI and HHPA is broken, which exposes the positively charged amino groups to the outside of the nanosystem. This increases the electrostatic interactions between the nanosystems and the cancer cells, which leads to a higher cellular uptake in 4T1 (triple negative breast cancer) tumours in mice, as can be seen by the NIR and colour-mapped MR images (Figure 5B and C). The MRI contrast effect is more pronounced at the tumour site after treatment with SPA-SPIO-IR780 nanoparticles than with pH unresponsive probe (SP-SPION-IR780), which can be explained by the pH-induced accumulation in tumours and by the large *r_2_* of these particles (254.09 mM^-1^ s^-1^ at 1.5 T versus 98.4 mM^-1^ s^-1^ for commercial CA Resovist®).

In terms of therapeutic efficiency, the treatment of mice with SPA-SPIO-IR780 nanoparticles followed by exposure to a NIR (780 nm) laser for 5 minutes led to a significant reduction of the tumour volume when compared to light excitation after treatment with PBS (tumour volume after 20 days was 255.2 mm^3^ and 2563.7 mm^3^, respectively). As such, this is a promising theranostic nanoplatform with remotely activated therapy that uses the responsiveness of the matrix polymer to enhance tumour uptake and the therapeutic outcome of PTT/PDT.

#### Dual pH and other stimuli-responsive smart theranostics

**Figure 6:**
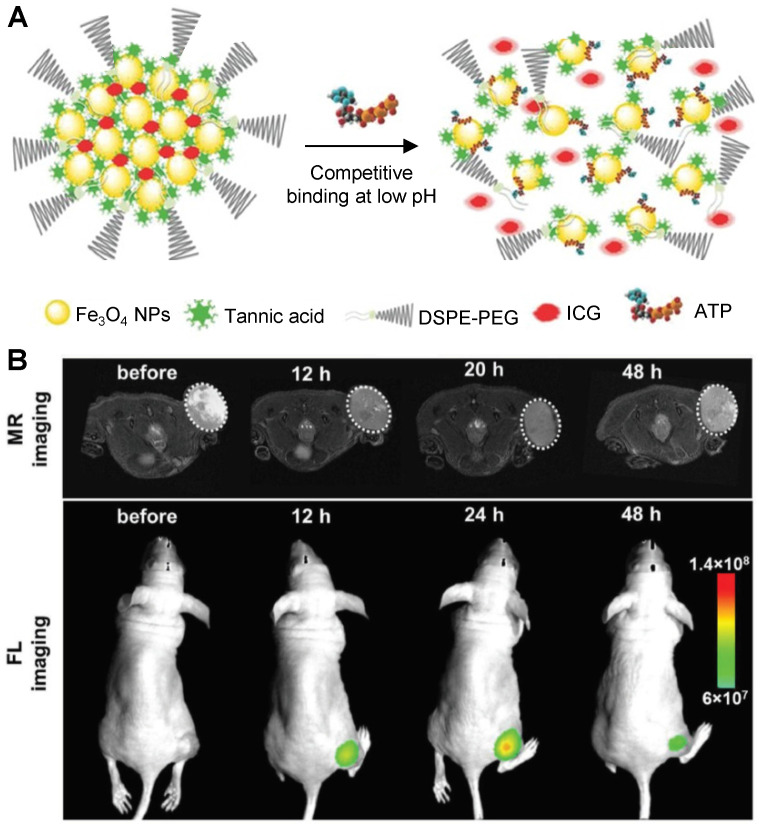
**A** Schematic illustration of the disassembly process suggested by Song *et al* 32. **B** *T_2_*-weighted MR images and fluorescence images of HepG2 tumour-bearing mice after intravenous injection of the Fe_3_O_4_@TA-PEG/ICG assemblies at 7.0 T. Adapted with permission from 32, copyright 2017, John Wiley and Sons.

Smart theranostics for PTT using MRI and FL have also been designed to exhibit ATP-responsive pH-facilitated disassembly (Figure 6A) 32. These nanoconstructs were based on iron oxide nanoparticles, tannic acid (TA) and indocyanine green (ICG) surrounded by polyethylene glycol-modified distearyl phosphatidyl ethanolamine (DSPE-PEG). These particles responded to ATP (upregulated in tumours) because it binds competitively to the surface of SPIONs by displacing the TA holding the assemblies together. They also responded to low pH, due to protonation of hydroxyl groups on TA and subsequent weakening of the coordination bonds between polyphenols in TA structure and metals from the iron-based particles, which loosens the assemblies. This mechanism brought an associated dramatic change in transverse relaxivity upon disassembly (from *r_2_*= 251.2 mM^-1^ s^-1^ at pH = 7.4 to *r_2_*= 13.68 mM^-1^ s^-1^ in the presence of 10 mM of ATP and at pH = 5.5), which was accompanied by a large “turn-on” of fluorescence signals (Figure 6B). The switch in the FL signals can be attributed to the loss of the quenching effect induced by the magnetite nanoparticles on ICG following disassembly of the constructs upon treatment with ATP. Furthermore, injection of the nanoparticles in HepG2 tumour bearing mice initially led to a slight darkening effect in MR images of the tumour region, due to the accumulation of a small number of intact assemblies at the target site (Figure 6). However, as the nanoplatforms encounter ATP in the cancerous tissues, the dark contrast effect decreased, due to ATP-induced disassembly. This also explains the increase in the fluorescence signals. Although the decrease in the brightness of the MRI contrast might be insufficient for a reliable diagnosis (as the clearance of the particles from the tumour will have the same effect on MR images), this mechanism allows for an easy detection of target sites using FL, since the signal intensity increases upon exposure to the stimuli. In terms of therapy, irradiation of treated mice with an NIR laser (808 nm, 1 W cm^-2^) led to a temperature increase of almost 20 °C in the tumours and translated into remarkable ablation of tumour size with no tumour recurrence post treatment for 16 days. This study is then an example of multi-stimuli responsive multimodal theranostics offering remotely activated therapy and environmentally switchable MRI and FL.

Ren *et al*. designed another multi-stimuli theranostic probe that they described as an “all-in-one” agent, since it combined seven distinct functionalities: MRI, Computed Tomography (CT) and PA imaging, and PTT, PDT, chemotherapy and RT 118. Their PEGylated doped metal chalcogenide nanoflowers were loaded with DOX (Bi-MS_X_-PEG/DOX NFs) and contained either bismuth (Bi), manganese (Mn) or molybdenum (Mo). Depending on the dopant, these nanoflowers could be used for MR/CT/PA-guided PTT/PDT/RT/chemotherapy and responded to pH, NIR light and X-rays. The nanoflowers were used for chemotherapy since they had a high DOX loading (DOX loading weight percentage of around 90% at 1:1 feeding mass ratio of DOX to NF) and released 80% of loaded DOX within 8 h in response to low pH (pH = 5.5) and exposure to a NIR laser. The petal structure of the nanoflowers enhanced their photoabsorption properties, translating into a high photothermal efficiency (54.7%) and thus making them useful as PTT effectors. Bi-MS_X_-PEG nanoflowers could also be used for PDT, as they contained MoS_2_ and Bi_2_S_3_ composites able to generate ^1^O_2_ upon exposure to an 808 nm laser. Nanoflowers with abundant Bi could also induce DNA damage to cells upon exposure to X-rays, since Bi has strong X- ray attenuation capacity and could thus be used for RT. In terms of imaging, these nanoflowers could work as *T_1_* agents for MRI (due to the presence of Mn^2+^ ions), as contrast enhancers for CT imaging (since Bi and Bi_2_S_3_ efficiently absorb ionising radiation), or as photoacoustic imaging agents (as MoS_2_ provides high and wide NIR optical absorbance). This novel “all-in-one” nanoplatform features both environmentally and remotely activated therapy and shows promising results in tumour ablation in mice. However, its efficacy as a multimodal imaging agent will greatly depend on the lifetime of the nanoconstructs in the bloodstream and inside cancer cells. Additionally, its pH activated therapy will depend on the NFs' ability to enter tumour cells, as the pH that they respond to (pH = 5.5) is lower than that of the tumour microenvironment.

pH-responsive MRI theranostic agents for chemodynamic therapy (CDT) have also been designed. CDT is a novel ROS-based cancer treatment that utilises iron-mediated Fenton chemistry to destroy tumour cells by converting endogenous H_2_O_2_ into toxic hydroxyl radicals (^●^OH). Xiao *et al.* synthesised nanoselenium-coated manganese carbonate-deposited iron oxide nanoparticles (MCDION-Se) that responded to the low pH of tumour microenvironments 128. At pH = 5.5, the nanoplatforms released Mn^2+^ ions, which increased the *r_1_* from 2.7 mM^-1^ s^-1^ (pH = 7.4) to 7.1 mM^-1^ s^-1^ (at 9.4 T). Following injection and pH-mediated release of Mn^2+^, the loaded nano-Se particles promoted the activation of superoxide dismutase (SOD) and consequent formation of H_2_O_2_ and ^·^OH radicals for CDT. Treatment of cervical tumour-bearing mice with MCDION-Se theranostics slowed down tumour progression but did not inhibit tumour growth, which suggests that a synergistic therapy approach could be considered in future works involving CDT to improve prognosis.

Small-molecule theranostics have found promising applications in cancer therapy 129-131; their structure is more straightforward than that of most nanoparticles and their synthesis is more reproducible. In 2016, Lee *et al.* reported a pH-responsive small-molecule MRI theranostic for cancer therapy 132. This system was equipped with a DOX prodrug conjugated to a Gd^3+^-texaphyrin complex *via* a pH-cleavable hydrazone linker. When intact, this complex was only weakly fluorescent, since DOX was quenched by the Gd^3+^-texaphyrin moiety. However, the fluorescence signal at 593 nm was switched from OFF to ON at low pH (pH = 5.0), due to the cleavage of the linker and separation of quencher and emitter. *In vitro* confocal microscope experiments with cancerous (A549 and CT26) and healthy (NIH3T3) cell lines showed that the Gd-texaphyrin-DOX complex was preferentially taken up by cancerous cells and was then hydrolysed inside lysosomes. Further studies indicated that this complex could also be used as a *T_1_* contrast enhancer (*r_1_*= 20.1 mM^-1^ s^-1^ at 1.4 T) that, when combined with FL, can be used to monitor of the uptake of DOX by cancer cells. This is a straightforward example of a small-molecule based theranostic with environmentally OFF/ON switchable fluorescence capabilities and environmentally activated therapy.

The response mechanisms of the discussed pH-activated theranostics have been summarised in Table 1.

### Redox responsive smart theranostics

The redox potential of neoplastic tissues is distinct from that of healthy tissues and also varies between extracellular and intracellular compartments 133. γ-L-glutamyl-L-cysteinyl-glycine, or GSH, plays a major role in the maintenance and regulation of the redox status of these tissues 134. GSH is synthesised in the cytosol and is present there and in subcellular organelles at a 2-10 mM concentration 133, 135. The concentration of GSH outside cells is much lower (2-10 µM) and this difference can be exploited in the design of smart theranostics. Small-molecule or nanosized theranostic agents can be designed to be highly stable in blood circulation and degrade and release their cargos in the GSH-rich tumour microenvironment or after internalisation in cancer cells. This can be achieved by integrating GSH reduction/oxidation-sensitive bonds in the theranostic agents, such as disulphide (S-S) bonds or reducible amine bonds 136.

**Figure 7:**
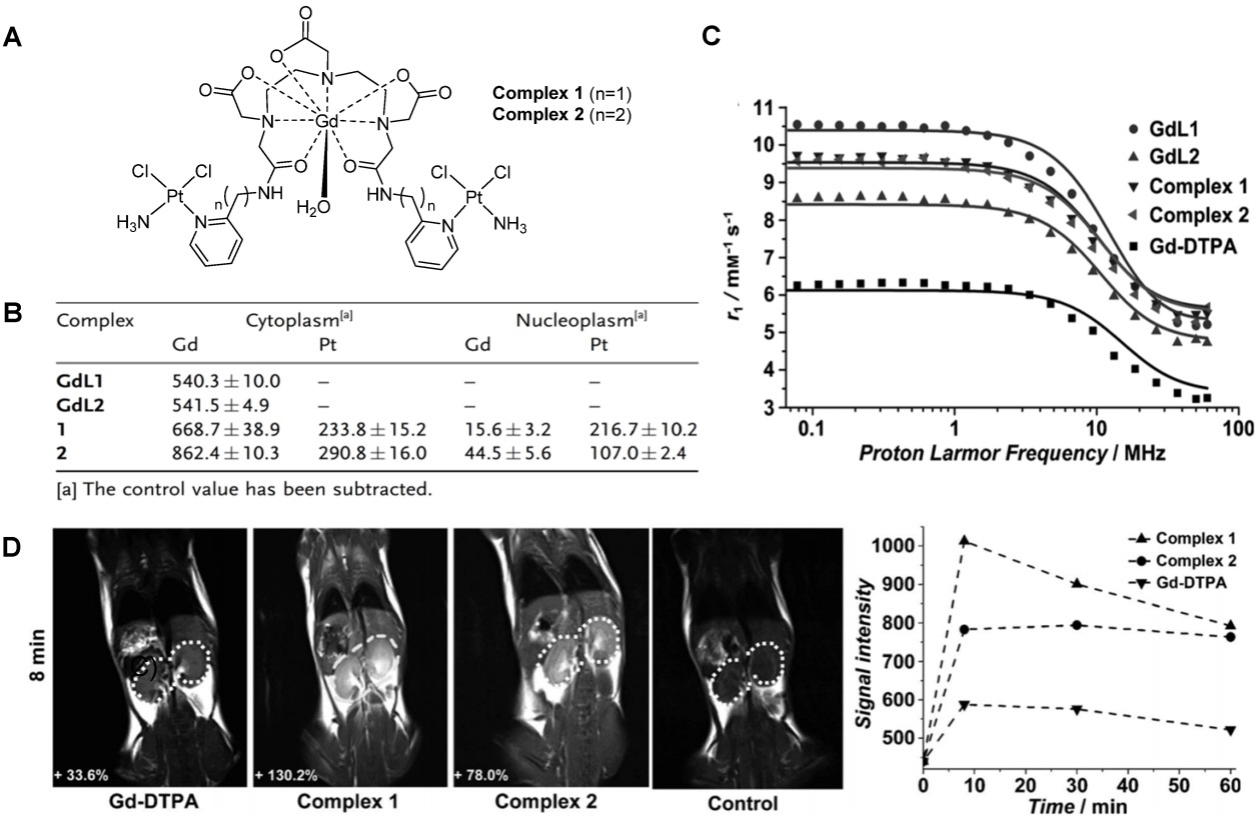
**A.** Structure of synthesized theranostic probes 1 and 2. **B.** Cellular uptake of complexes 1 and 2 and Gd-complexes GdL1 and GdL2 (without the platinum moiety) in the cytoplasm and nucleus of HeLa cells measured by ICP-MS. **C.** NMRD profiles of synthesized complexes and Gd-DTPA at different magnetic-field-strengths. **D.** *T1*-weighted MR images of mice at 8 min after the injection of complexes 1 and 2, and Gd-DTPA (0.1 mmol kg^-1^), at 3.0 T, and the corresponding MR signal-decay curves. Adapted with permission from 137, copyright 2014 John Wiley and Sons.

Small-molecule theranostics have also been designed to respond to redox agents in the tumour microenvironment. In 2014, Zhu *et al.* reported the synthesis and *in vitro* and *in vivo* validation of a theranostic Gd-Pt probe that could be used to monitor the biodistribution of the platinum-based chemotherapy drug in real time, and presented cytotoxicity values similar to those of cisplatin, even when used at imaging concentrations (Figure 7A) 137. Since the therapeutic mechanism of platinum complexes mostly involve translocation into the nucleus and binding to DNA, the authors studied the cellular uptake of the theranostic probe in HeLa cells by inductively coupled plasma mass spectrometry (ICP-MS, Figure 7B). From the results, it is possible to see that the complexes containing a platinum moiety successfully entered cells, as Gd(III) and Pt(II) were both detected in the cytoplasm. Then, complexes 1 and 2 underwent redox-induced dissociation and the platinum moieties migrated and accumulated in the nucleus (Pt:Gd ratio in the nucleoplasm for complex 1 is around 14:1). However, complexes GdL1 and GdL2 did not accumulate in the cytoplasm or nucleus to the same extent, which shows that the platinum moieties may facilitate the cellular uptake of the probe. The relaxivity of the probes was also analysed by proton nuclear magnetic relaxation dispersion (NMRD) and results showed that complexes 1 and 2 (*r_1_*= 6.3 mM^-1^ s^-1^) had higher relaxivity than commercially available Gd-DTPA (*r_1_*= 4.3 mM^-1^ s^-1^, Figure 7C). This enhancement of the MR signal was also visible *in vivo*, in mice that were injected intravenously with either complex 1, 2 or Gd- DTPA (Figure 7D). Although the *in vitro* results seemed to indicate that complex 2 would accumulate to a higher extent inside cells, the *in vivo* results showed that complex 1 induced a brighter MRI signal in the kidneys, possibly due to different internalisation kinetics *in vivo*. As such, these environmentally responsive chemotherapy complexes could be used to monitor the distribution of platinum drugs and assess their therapeutic response in real time. Other Gd-Pt complexes have been tested as GSH-responsive theranostics for cancer following the same rationale 138, 139.

**Figure 8:**
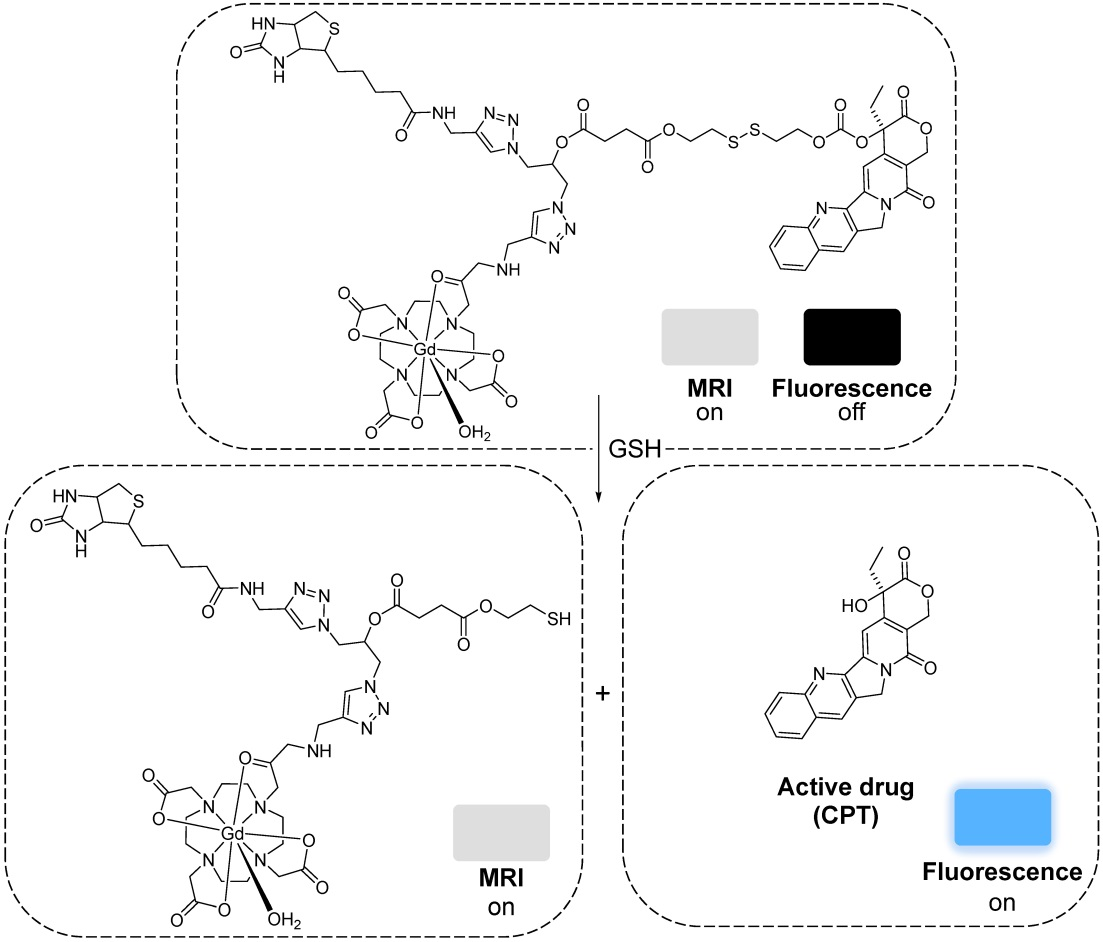
The mechanism of the release of CPT from the gadolinium theranostic agent 140.

More recently, a target specific redox-responsive small-molecule theranostic prodrug containing a gadolinium contrast agent was synthesized and biologically tested 140. This theranostic agent contained: 1) Gd-DOTA as a contrast agent, 2) biotin as a targeting agent that binds to overexpressed biotin receptors in cancer cells, 3) camptothecin (CPT) as an anticancer drug and fluorescent probe, and 4) a disulphide linker that responds to the reductive environment inside cancer cells (Figure 8). The authors performed *in vitro* and *in vivo* studies that showed that the MRI signal gradually intensified after treatment of A549 cells or mice bearing A549 tumours with the probe. *T_1_*-weighted images showed that the prodrug selectively bound to tumour cells. Analysis of the tumours' growth also showed that the prodrug was not only being taken up by cancer cells but also that it was being metabolized in response to the reducing environment, thus releasing the anticancer drug specifically inside the tumour. This kind of small-molecule design, although synthetically challenging, has advantages associated with its simplicity, including a better understanding and modulation of the mechanism of drug release and, in some cases, rapid renal clearance. However, its simplicity also makes it difficult to integrate multiple functions in a single agent. Additionally, it might be more challenging to surpass the detectable contrast enhancement threshold, due to the often less efficient tumour cell uptake and accumulation 141. Alternatively, redox-responsive nanosystems have also been designed with different nanomaterials: mesoporous silica nanoparticles (MSNs) 142, 143, carbon nanomaterials 144, 145, gold nanomaterials 105, 146 and others 133, 147-152. By incorporating a magnetic component, some of these nanoparticles could be used as MRI theranostics for cancer therapy.

For example, Zhu *et al.* designed a theranostic system by binding a platinum anticancer prodrug to poly(ethylene glycol) coated Fe_3_O_4_ SPIONs (PEG-SPIONs) 147. The conjugation with PEG molecules ensured that the nanoparticles had improved chemical and colloidal stability, biocompatibility and pharmacokinetics. The use of a cisplatin prodrug, HSPt, improved the cytotoxicity of the system towards cancer cells. Usually, cisplatin is chelated by GSH to form Pt-GS-Pt adducts that are unable to bind to DNA. However, in this case, the authors reported limited Pt(II) detoxification by GSH, as the intermediate adduct Pt-GS, which still retains some DNA binding abilities, was detected at much higher concentrations than Pt-GS-Pt. It was also reported that treatment with these nanoparticles induced depletion of GSH levels of up to 48.7% (for A549 cells). The participation of GSH in the reduction of Pt(IV) to Pt(II) complexes seemed to contribute to the considerable depletion of GSH levels, which resulted in a less efficient detoxification process when compared to cisplatin and thus to an enhanced cytotoxic effect of these Pt(IV) complexes.

**Figure 9:**
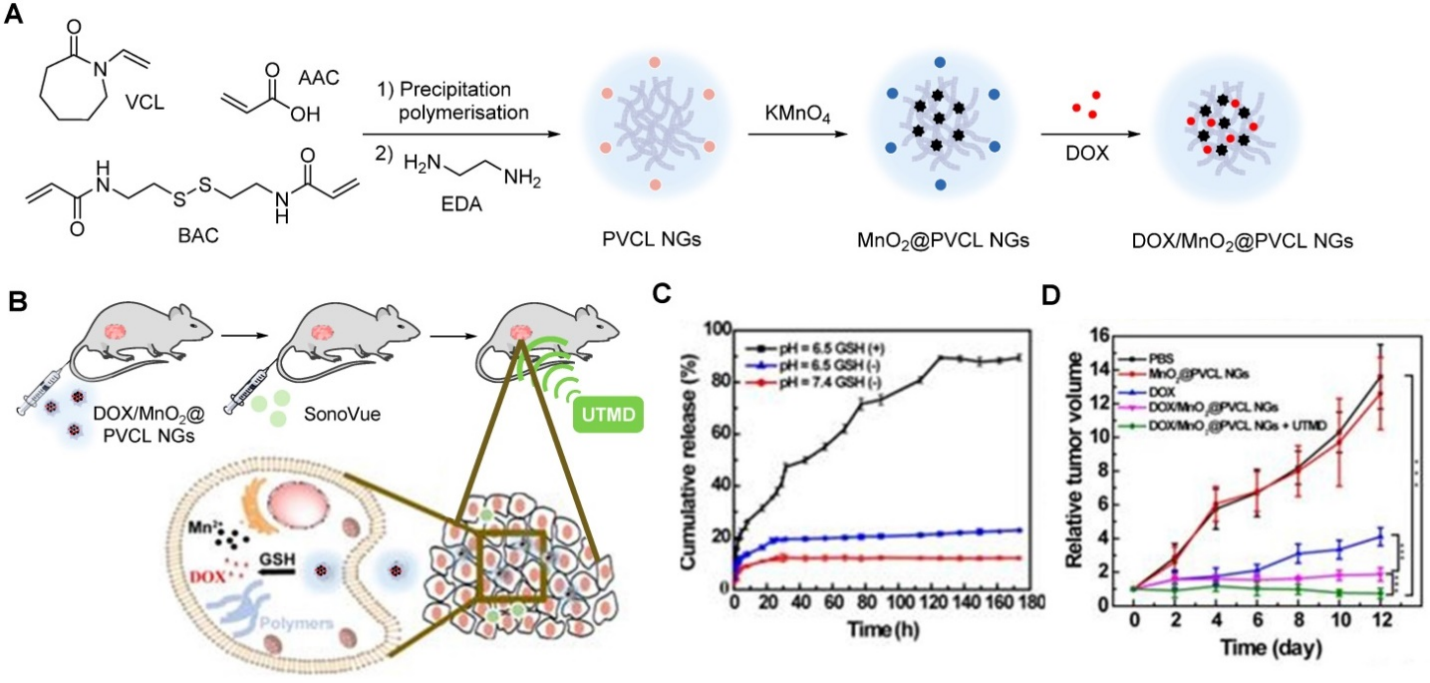
** A** Synthetic route for the fabrication of DOX/MnO_2_@PVCL NGs. **B** Schematic illustration of the utilization of DOX/MnO_2_@PVCL NGs for UTMD-promoted delivery of NGs for MRI-guided cancer chemotherapy. **C** DOX release profile from DOX/MnO2@PVCL NGs at pH = 7.4 or 6.5 in the presence or absence of GSH (10 mM). **D** Tumour growth curves after different treatments (n = 5). Tumour volumes were normalized to their initial values. Adapted with permission from 148, copyright 2020 Ivyspring International Publisher.

Manganese oxide nanoparticles can also be used as redox responsive *T_1_* contrast agents in nanocarriers. A redox-responsive hybrid nanosystem composed of DOX and poly(N-vinylcaprolactam) (PVCL) co-loaded nanogels (NGs) and with MnO_2_ nanoparticles was designed for MRI-guided and ultrasound-targeted microbubble destruction (UTMD) promoted chemotherapy 148. These DOX/MnO_2_@PVCL NGs were synthesised by a precipitation polymerisation procedure that was followed by loading with MnO_2_ nanoparticles through oxidation with permanganate (Figure 9A). DOX was added *via* physical encapsulation and Mn-N coordination to afford 106.8 nm nanoparticles. The NGs' ability to release DOX in response to redox species like GSH was evaluated. Results demonstrated that after 7 days, only 10% of DOX had been release in a buffer at pH = 7.4 without GSH (Figure 9C). At the same time point, 20% was released in a buffer without GSH at pH = 6.5. Importantly 89.5% of DOX was released from the NGs after 7 days incubation in a medium containing 10 mM of GSH at pH = 6.5. The drug release was potentiated by GSH-mediated cleavage of S-S bonds within the nanogels, which induced swelling of the nanoparticles and freed DOX. The relaxometry of the DOX/MnO_2_@PVCL NGs was also dependent on GSH, since *T_1_* enhancer Mn^2+^ was released in the presence of the redox agent. The *r_1_* of the NGs increased from 0.04 mM^-1^ s^-1^ to 8.33 mM^-1^ s^-1^ at 0.5 T in solutions with 10 mM of GSH (*r_1_* of Magnevist at 0.5 T is 4.56 mM^-1^ s^-1^). *T_1_*-weighted MRI showed adequate signal enhancement properties for these nanogels. The antitumoral effect of the theranostic systems was evaluated in the presence and absence of UTMD and compared to the efficacy of DOX by itself (Figure 9D). UTMD can be used to enhance chemotherapy since it uses sonoporation to increase tumour capillary permeability and endocytosis. Results showed that the DOX/MnO_2_@PVCL NGs theranostic platforms with environmentally activated therapy and switchable contrast agents, exhibited enhanced therapeutic efficacy when combined with UTMD in comparison to treatment with DOX or NGs only. This type of design, however, poses the same concerns regarding neurotoxicity of free Mn^2+^ ions.

**Figure 10:**
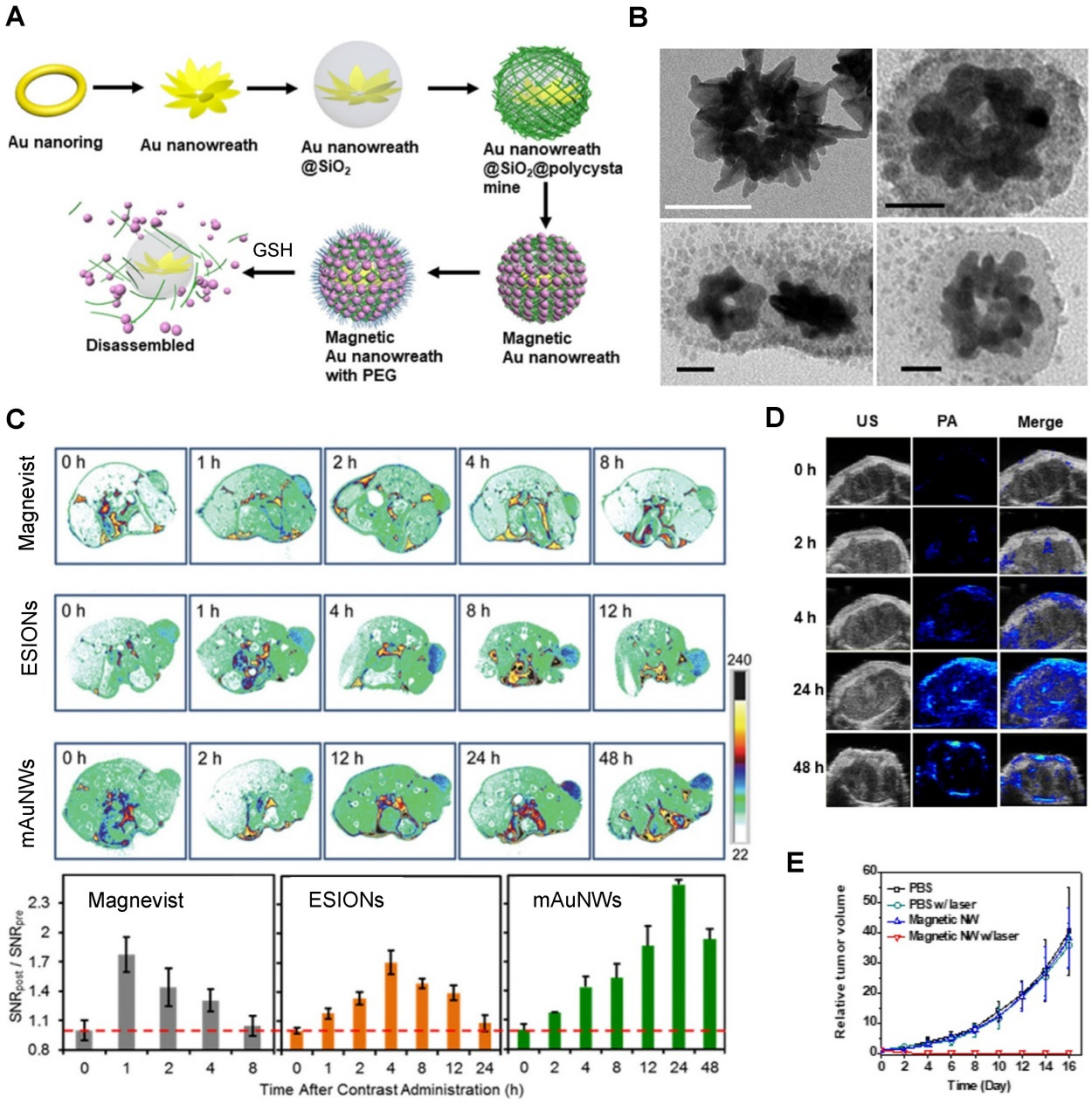
**A** Schematic illustration of the synthesis of magnetic gold nanowreaths. **B** Representative TEM images of AuNWs (top left), magnetic AuNWs before incubation with GSH (top right), magnetic AuNWs after 30 min incubation with 10 mM GSH (bottom left) and magnetic AuNWs after 2 h incubation with 10 mM GSH(bottom right); scale bar: 50 nm. **C** *T_1_*-weighted images of U87MG tumour-bearing nude mice and corresponding quantificational analysis of the tumour signals after intravenous injection of 5.0 mg/kg of Magnevist, ESIONs or magnetic AuNWs. The signal enhancement in tumours for different contrast agents at different time points after injection were quantified as the division of signal-to-noise ratio (SNR) of tumours at different time points post injection by the SNR of tumours at 0 h. **D** *In vivo* PA and PTT with magnetic AuNWs showing US, PA, and merged images of tumour before injection and at 2, 4, 24, and 48 h after intravenous injection of magnetic AuNWs upon irradiation by an 808 nm pulsed laser. **E** Relative tumour volume after different treatments and irradiation with an 808 nm CW laser at 0.75 W cm^-2^. Adapted with permission from 105, copyright 2018 American Chemical Society.

The redox responsiveness of gold nanomaterials has also been explored. Liu *et al.* designed magnetic gold nanowreaths (AuNWs) that could be used for redox-responsive MR, PA and PTT 105. These nanowreaths were comprised of an inner core of AuNWs surrounded by a layer of silica and a shell of PEG-conjugated ESIONs. The inner core of the nanoparticles was synthesised by Au-nanoring-mediated growth, followed by introduction of a silica shell and then layer-by-layer self-assembly of polymers and ESIONs (Figure 10). Finally, PEG was conjugated to the ESIONs to afford magnetic AuNWs with an overall diameter of 100.7 nm.

These platforms were found to be good PTT effectors and showed interesting MRI properties. When the magnetic nanowreaths were intact, with ESIONs, (usually good *T_1_* CAs) compacted onto the surface, the overall *r_1_* of the complex was relatively low (*r_1_*= 1.1 mM^-1^ s^-1^ at 7 T) and the *r_2_* was high (*r_2_*= 198.6 mM^-1^ s^-1^ at 7 T). This resulted in darker contrast in the MR images, due to the low surface-to-volume ratio of the assembled particles. However, in the presence of GSH (10 mM), the *r_2_* decreased to 33.9 mM^-1^ s^-1^ and the *r_1_* increased, to 3.2 mM^-1^ s^-1^, which allowed for a *T_1_* switch from “on” to “ON” in the presence of GSH. This effect was caused by the cleavage of disulphide bonds in the polymers attached to the silica layer, which allowed for the release of ESIONs from the nanoconstructs that could be observed in TEM images (Figure 10B). The MR images of U87MG tumour bearing mice show that the injection of magnetic AuNWs produces a *T_1_* signal enhancement, which means that the probe is being cleaved inside GSH-rich tumour cells (Figure 10C). Additionally, the brightest *T_1_* signal enhancement (2.2 times enhancement when compared to pre-injection signal) of magnetic AuNWs occurs after 24 h, while Magnevist and ESIONs induce maximum enhancement of 1.8 and 1.7 times the pre-injection signal at 1 and 4 h, respectively. PA was also used and confirmed that the signal enhancement gradually increases until 24 h post injection (Figure 10D). This work also shows that these magnetic AuNWs allowed for the effective removal of subcutaneous tumours through PTT, since the gold branches, small junctions, and central holes of the nanowreaths improved the absorbance and photothermal properties of the gold nanoconstructs (Figure 10E). This is then an example of a multifunctional and multimodal theranostic that combines remotely activated therapy with environmentally switchable imaging agents that show a distinct contrast enhancement in GSH-rich tumour cells.

Photosensitiser nanoassemblies (NP-RGDs) have also been reported as GSH and light-responsive dual modal MRI/FL theranostic platforms for PDT 153. These nanoparticles were prepared *via* self-assembly of an NIR photosensitiser pheophorbide (PPa) for FL and PDT, a GSH-cleavable linker, a fluorophore to facilitate self-assembly, a paramagnetic Gd-DOTA chelate for MRI and a cyclic RGD peptide for α_ν_β_3_ targeting. *In vivo* studies showed that the NP-RGD nanoparticles worked as *T_1_* contrast agents (*r_1_*= 20.0 mM^-1^ s^-1^ at 0.5 T) and selectively accumulated in U87MG tumours, leading to a 61% signal enhancement in tumours 2 h after injection, a 9-fold increase when compared to untargeted nanoassemblies. Following entry into tumour cells, the disassembly of the nanoparticles, which had quenched fluorescence and PDT abilities, was triggered by disulphide reduction by GSH. A fluorescent product and PPa-SH, a hydrophobic photosensitiser, were then formed and the fluorescence at 547 nm was increased by 77-fold, which translated into the FL images. PPa-SH was then bound to endogenous albumin, which afforded prolonged retention in tumours, more efficient ^1^O_2_ generation for PDT, and consequently more efficient ablation of tumours.

Lin *et al.* designed GSH-responsive MRI theranostics that could be used for CDT 154. Since the efficiency of CDT is diminished by the elevated levels of detoxifying molecules like GSH present in tumour microenvironments or cancer cells, these researchers designed MnO_2_-functionalised mesoporous silica nanoparticles (MS@MnO_2_ NPs) with GSH depletion properties. These theranostics had environmentally switchable imaging abilities, as the *r_1_* of the nanoplatforms increased 13-fold upon GSH-mediated release of Mn^2+^ ions from the NPs' shell. This OFF/ON switch was also observable *in vivo* in *T_1_*-weighted images, where a significant contrast enhancement was observed in U87MG tumours up to 24 h post injection of the MS@MnO_2_ NPs. *In vivo* studies were also used to evaluate the CDT efficiency of the theranostic platforms and showed that tumour growth was significantly supressed, but not inhibited, after injection with the NPs. Modified CPT-loaded nanoparticles (MS@MnO_2_-CPT NPs) were also tested and displayed tumour growth inhibition at a reduced Mn dose. This example suggests once more that CDT on its own can decrease tumour growth rates, but it is in combination with other treatment modalities where CDT shows the most promising effects.

The response mechanisms of the discussed pH-activated theranostics have been summarised in Table 2.

### Enzyme responsive smart theranostics

Enzyme-responsive theranostics have also been explored, to take advantage of the altered expression levels of enzymes in pathological conditions, including cancer 155. Researchers have been interested in using enzymes as an endogenous stimulus for drug release because enzymatic reactions are fast, highly efficient, and very specific and selective. Different theranostic agents have been designed to respond to matrix metalloproteinases (MMPs) 156, cathepsins 157, 158, cysteine caspases, phospholipases, or hyaluronidases, for example 29, 159.

Santra *et al.* developed a convenient methodology that allowed for the synthesis of an esterase-sensitive theranostic agent 160. The method included co-encapsulation of an anticancer drug, taxol, and NIR dyes inside hydrophobic pockets of a polyacrylic acid (PAA) coated SPION. This complex was further modified by “click chemistry” to include folate as a targeting agent towards cancer cells. These nanoparticles selectively released taxol and NIR dyes in an esterase-dependent manner or in acidic conditions. Although the mechanism by which the nanosystems release taxol in response to the enzyme is not fully elucidated, this could be an easy and scalable strategy to synthesize targeted theranostics with enzyme activated therapy and switchable fluorescence signals.

**Figure 11:**
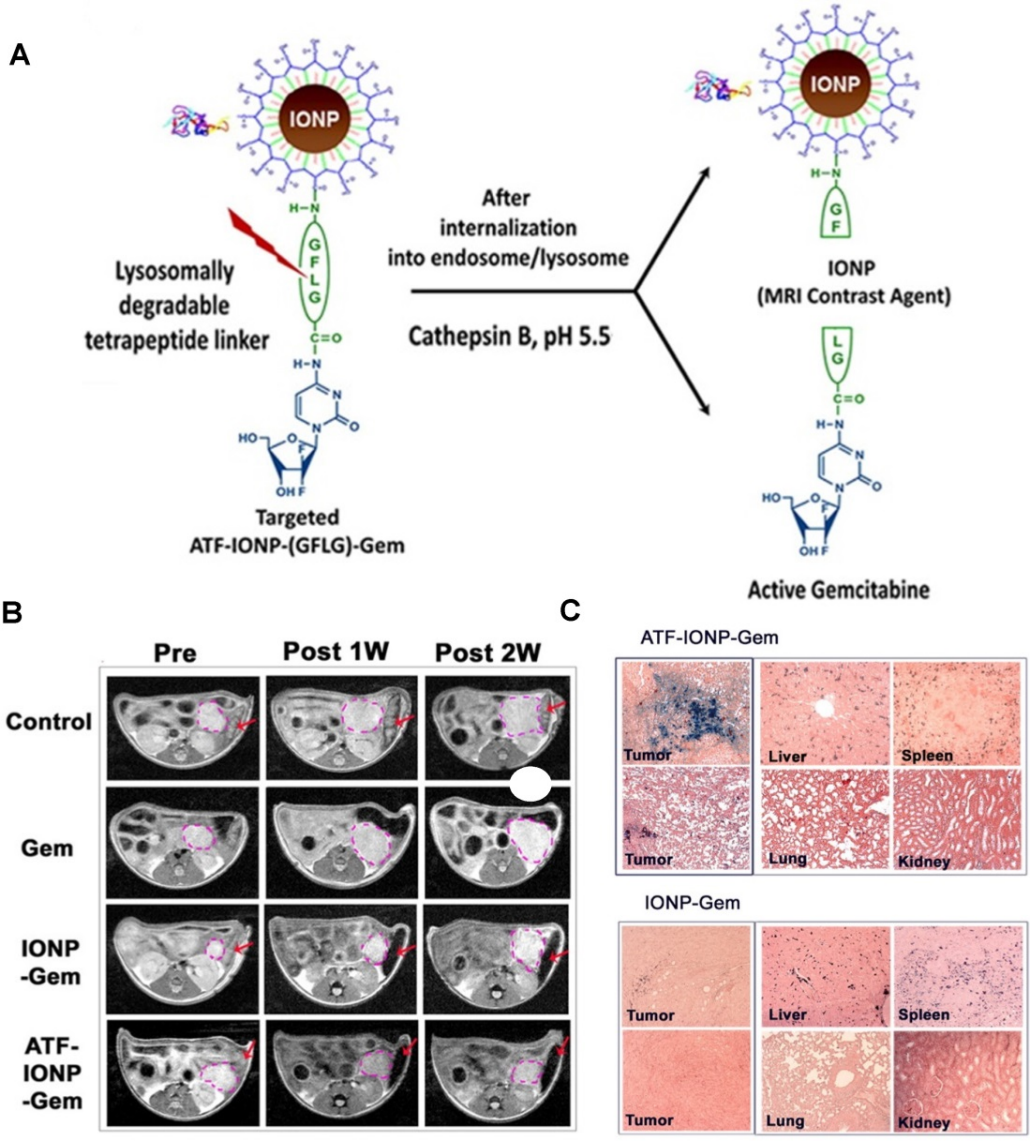
**A.** Schematic diagram of Gem release from ATF-IONP-Gem. **B.** Axial *T_2_*-weighted MR images of the tumour-bearing mice before and one week and two weeks after receiving theranostic nanoparticles, at 3.0 T. Post-treatment images were obtained 48 h following the second (1 week) and fourth (2 weeks) injection. The location and size of the cancer lesions (pink dotted circles) can be seen in the MR images. Red arrows indicate the MRI contrast change in the spleen. **C.** Biodistribution of ATF-IONP-Gem and non-targeted IONP-Gem following systemic treatments. Tumour and normal tissue sections obtained from mice at the end of the five treatments were stained with Prussian blue staining. Blue: IONP positive cells; red: Nuclear Fast Red background staining. Adapted with permission from reference 157, copyright 2013 American Chemical Society.

Lee *et al*. also synthesised a SPION-based enzyme-sensitive MRI theranostic agent, in this case responsive to cathepsin B 157. They engineered urokinase plasminogen activator receptor (uPAR)-targeted magnetic iron oxide nanoparticles (IONPs) that delivered chemotherapy drug gemcitabine (Gem) to uPAR-expressing tumour and stromal cells, such as pancreatic cancer cells. These ATF-IONP-Gem nanoconstructs were prepared by conjugation of the targeting vector, an amino-terminal fragment (ATF) peptide of the receptor-binding domain of natural ligand uPA, to the IONPs. Gem was also conjugated to the nanoparticles through a tetrapeptide linker that can be cleaved by lysosomal cysteine protease, cathepsin B, which is overexpressed in pancreatic cancer cells (Figure 11A). HPLC analysis showed that the release of Gem from these nanoconstructs was dependent on the presence of cathepsin. Indeed, these studies showed that 82% of Gem was released from ATF-IONP-Gem nanoparticles in response to cathepsin and in mildly acidic conditions (pH = 5.5). MIA PaCa-2 tumour-bearing mice were treated with 2 mg kg^-1^ of Gem alone, non-targeted IONP-Gem or ATF-IONP-Gem twice per week, for a total of five times. The antitumor effect of these treatments was evaluated *in vivo* and results showed that ATF-IONPs led to 50% tumour growth inhibition, which is higher than the inhibition observed after injection with Gem or IONP-Gem, 23% and 30%, respectively. This implies that the use of the targeting agent positively affects the therapeutic outcome.

This targeted delivery was imaged using *T_2_*-weighted MRI. These nanoplatforms present a *r_2_* of 195 mM^-1^ s^-1^ at 3 T, which is much higher than that of Endorem® (45 mM^-1^ s^-1^ at that field strength) 161. *In vivo* MR images (Figure 11B) show that the targeted theranostic agents contributed to a decrease of 24% in the MRI signal at the tumour site following systemic delivery, which makes it possible to image drug delivery and accumulation in cancerous tissues. The accumulation of ATF-IONP-Gem nanoparticles was also confirmed by histological studies, which show specific accumulation of the targeted theranostic platforms in tumours, while no significant accumulation in healthy tissues was observed (Figure 11C).

**Figure 12:**
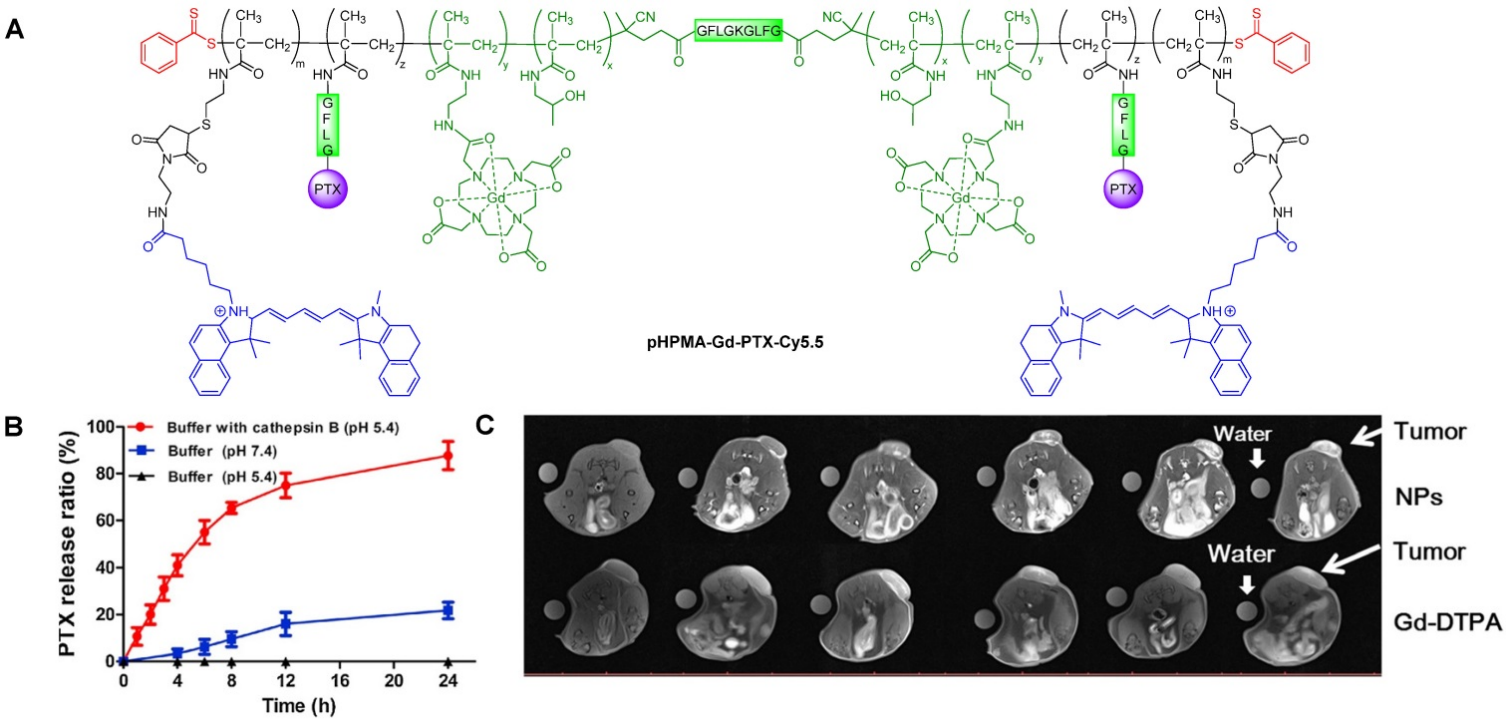
** A.** Structure of pHPMA-Gd-PTX-Cy5.5 conjugate. **B.** *In vitro* drug release profiles of PTX-conjugated nanoparticles after incubation in pH = 5.4 McIlvaine's buffer with cathepsin B (2.8 µM), pH = 5.4 McIlvaine's buffer without cathepsin B, and pH = 7.4 PBS without cathepsin B at 37 °C. **C.** MR axial imaging of tumours in tumour-bearing mice intravenously injected with Gd-DTPA or nanoparticles at predetermined time points (0.08 mmol Gd(III) kg^-1^ mouse), at 3.0 T. Adapted with permission from reference 158, copyright 2018 Elsevier.

Cai *et al.* also designed a cathepsin B-sensitive theranostic agent, but it was small-molecule polymer-based. They prepared novel amphiphilic biodegradable conjugates, pHPMA-Gd-PTX-Cy5.5, made of a gadolinium chelate as a *T_1_* MRI agent, chemotherapy drug paclitaxel (PTX), fluorescence dye cyanine 5.5 (Cy5.5) and a cathepsin B-sensitive GFLG peptide (Figure 12A) 158. First, a pHPMA-DOTA-PTX copolymer was synthesised by a two-step reversible addition-fragmentation chain transfer (RAFT) polymerization. These copolymers were then labelled with Cy5.5 through a click reaction, which was followed by a complexation reaction between Gd^3+^ and the DOTA chelators. This afforded pHPMA-Gd-PTX-Cy5.5 as 92 kDa nanoparticles after hydrophobic interaction-induced self-assembly of the polymers. The release of PTX from the conjugates was evaluated with reverse phase HPLC, in the presence and absence of cathepsin B, at different pH values (Figure 12B). Results showed that up to 90% of PTX was released within 24 h in a medium containing cathepsin B (2.8 µM) at pH = 5.4, which was much higher than the release of PTX without the lysosomal enzyme (20% release after 24 h). The nanoparticles were also evaluated as *T_1_* MRI contrast agents. The pHPMA-Gd-PTX-Cy5.5 constructs had *r_1_*= 12.9 mM^-1^ s^-1^ at 1.5 T, which was significantly higher than the relaxivity of commercial Gd-DTPA at that magnetic field strength (2.4 mM^-1^ s^-1^). The high efficiency of these nanoparticles as MRI CAs was also observed during *in vivo* MRI of mice bearing 4T1 breast carcinoma xenografts, at 3 T (Figure 12C). When compared to Gd-DTPA, pHPMA-Gd-PTX-Cy5.5 platforms exhibited a more gradual but more intense signal increase at the tumour site that lasted up to 24 h after injection. This showed that the nanoparticles have extended blood circulation and accumulate preferentially at tumour sites. ICP-MS studies indicated that the nanoparticles' route of excretion mainly involved the liver and spleen, since there was an accumulation of 26% and 13% per gram, respectively, in these organs after 24 h, which decreased to 11.4% g^-1^ and 3.5% g^-1^ at 96 h post injection. The pHPMA-Gd-PTX-Cy5.5 conjugate based nanoparticles provided significantly enhanced antitumoral properties, since they inhibited tumour growth by 95.5% on average (Figure 12D) versus only 16.7% of free PTX. This is a successful example of how small molecules can be conjugated into a larger polymer-based nanoparticle with enhanced blood circulation, enzyme-responsive drug release mechanism, increased efficacy as a *T_1_* contrast agent and improved anticancer properties.

Recently, the same group of researchers designed a similar stimuli-responsive polymeric nano-agent with enhanced MR, fluorescence and antitumoral efficacy following the same rationale 28.

Shi *et al.* designed an MMP-2 activated Gd-doped CuS nanoprobe (T-MAN) that could be used for dual modal MR/FL imaging of gastric tumours 162. The nanoparticles were formed by covalent modification of Gd-doped CuS micellar nanoparticles with a cRGD peptide and an MMP-2-cleavable fluorescent substrate. These nanoplatforms had high relaxivity values (60 mM^-1^ s^-1^ per Gd at 1 T), which, coupled with the dual targeting strategies employed in the NPs (targeting of α_ν_β_3_ integrins through RGD peptide and targeting of tumour microenvironment *via* addition of an MMP-2 substrate), resulted in the highest uptake of T-MAN in gastric tumours 12 h post injection (~23.4% ID% g^-1^) and translated in a significant *T_1_* signal enhancement. The efficiency of these platforms as PTT effectors was also tested in MKN45/Luc gastric-tumour bearing mice. T-MAN nanoparticles had a high photothermal conversion efficiency (70% following treatment with 808 nm laser, 0.85 W cm^-2^ for 10 min). Exposure of mice to a 808 nm laser after injection of T-MAN theranostics led to a more significant temperature increase in the stomach of tumour-bearing mice (25.3 to 57.4 °C) when compared to healthy subjects (25.2 to 33.9 °C), which suggests that these nanoparticles work well as targeted MRI/FL theranostics with environmentally switchable FL imaging and remotely activated PTT.

The response mechanisms of the discussed pH-activated theranostics have been summarised in Table 3.

### Temperature responsive smart theranostics

Temperature-responsive theranostic agents usually exhibit changes in the physical conformation of their materials, like swelling, in response to an increase in temperature, which subsequently triggers the drug release 35, 41, 163, 164.

**Figure 13:**
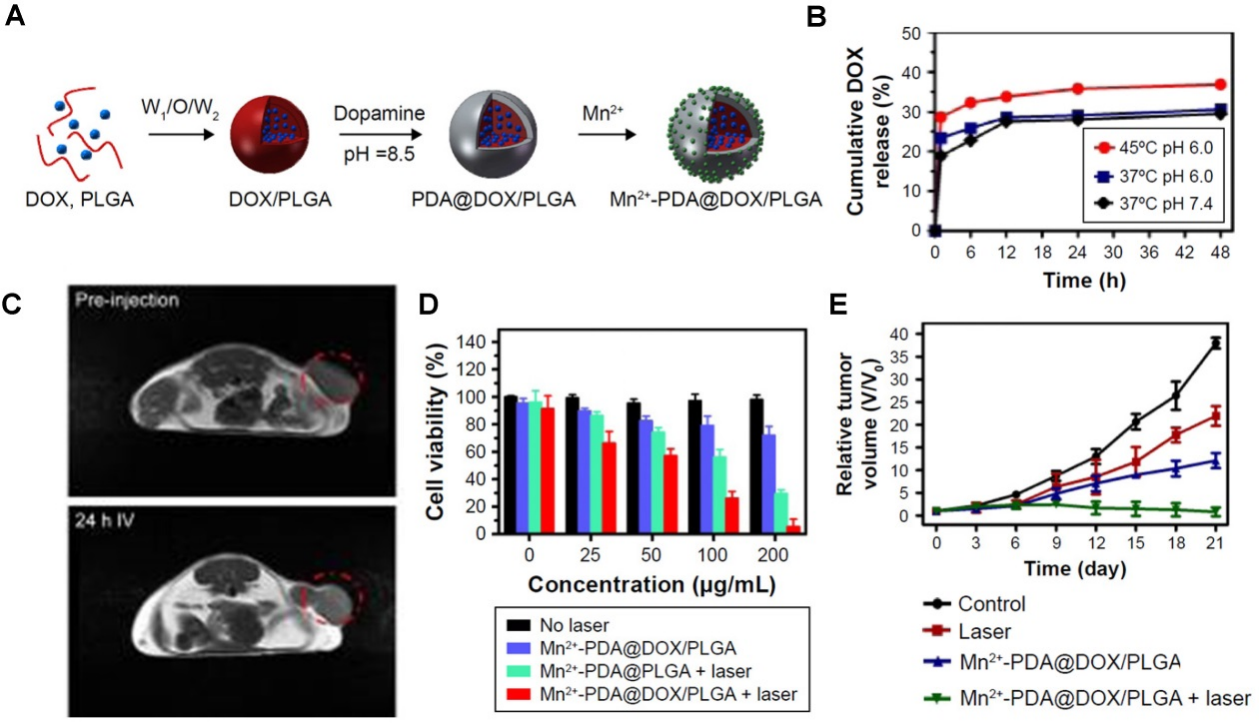
**A** Schematic diagram of synthesis process of Mn^2+^-PDA@DOX/PLGA nanoparticles. **B.** DOX release percentage from Mn^2+^-PDA@DOX/PLGA at varied pH values and temperatures. **C.** *In vivo T_1_*-weighted MR images of a mouse taken before injection (upper) and 24 h post intravenous injection (bottom) with Mn^2+^-PDA@PLGA, at 3.0 T. The red circle is the tumour region of the mice. **D.** Relative viabilities of CT26 cells incubated with various concentrations of Mn^2+^-PDA@PLGA and Mn2+-PDA@DOX/PLGA nanoparticles with or without an 808 nm laser irradiation (1.0 W cm^-2^) for 10 min. **E.** Tumour growth curves of different groups after treatment. The tumour volumes were normalized to their initial sizes. Adapted with permission from 35, copyright 2017 Dove Medical Press.

Xi *et al.* prepared DOX-loaded poly(lactic-co-glycolic acid) (PLGA) nanoparticles modified with polydopamine (PDA) that included Mn^2+^ ions coordinated on the surface of the nanoparticles (Figure 13A) 35. These Mn^2+^-PDA@DOX/PLGA nanoplatforms were designed as temperature responsive chemotherapy agents and PTT photosensitisers for MRI, due to the presence of DOX on the core and PDA on the surface of the nanoparticles, respectively.

The release of DOX was analysed by UV-vis spectroscopy and inductively coupled plasma atomic emission spectroscopy (ICP-AES) and showed that the drug release is triggered by increased temperature originating from the absorption of a NIR light by PDA (Figure 13B). As such, this theranostic platform could be considered light responsive, and not temperature responsive, but it was classified as the latter since the drug release is potentiated by the temperature increase, which causes the loosening of the PLGA mesh, not by the laser irradiation itself. *In vivo T_1_*-weighted MR images show a slight contrast enhancement 24 h post injection with the Mn^2+^-PDA@PLGA nanoparticles, due to the presence of Mn^2+^ ions on the surface and accumulation at target sites (Figure 13C). The therapeutic efficacy of these particles was confirmed in *in vitro* and *in vivo* assays, which showed a synergic effect between chemotherapy and PTT when mice were exposed to an 808 nm laser following treatment with DOX-loaded nanoparticles (Figure 13D and E).

**Figure 14:**
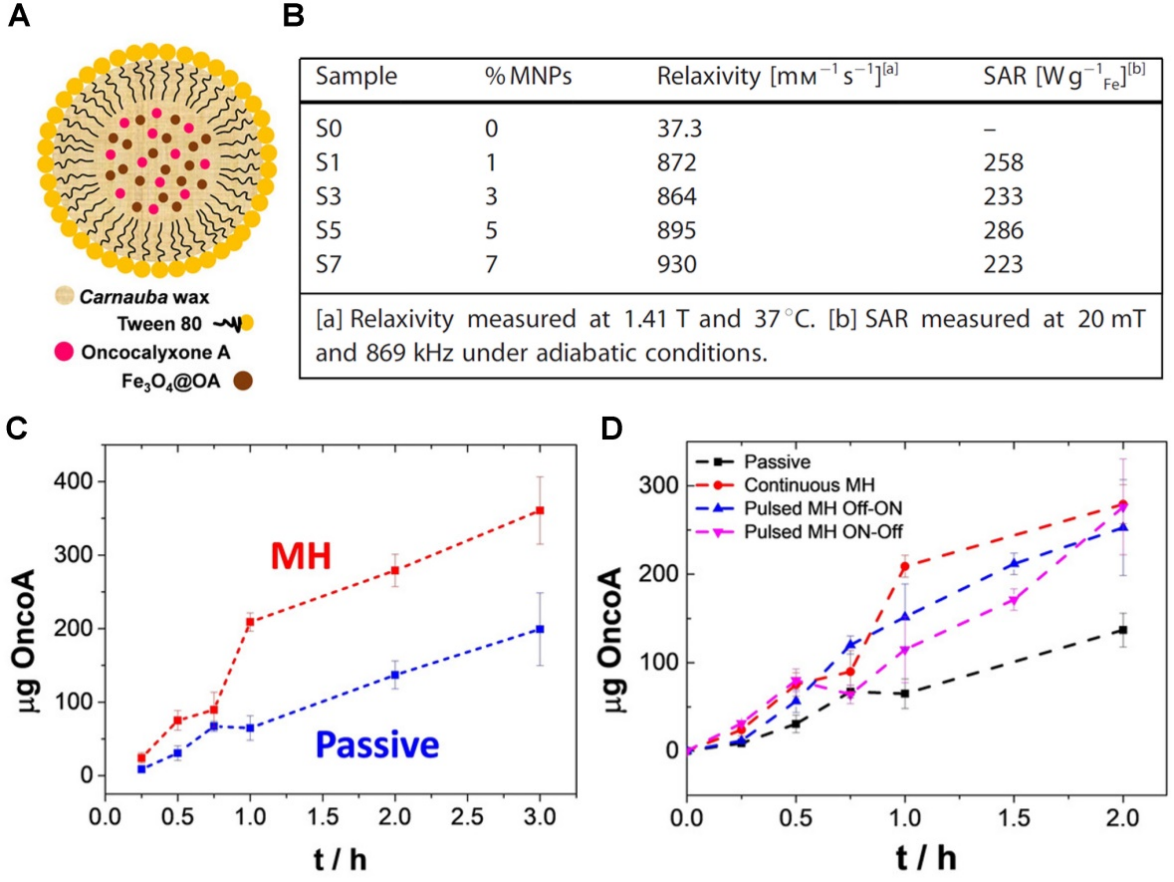
**A.** Schematic representation of the mWNVs. **B.** Relaxometric and MH properties of the mWNVs. **C.** Comparison of passive *versus* MH-induced release (24 mT and 523 kHz, 2 h) of Onco A from S7-Onco A during the first 3 h. **D.** Comparison of the cumulative release of Onco A from S7-Onco A under different conditions: black, room temperature passive release; red, continuous MH-induced release; blue, pulsed Off-ON MH-induced release; pink, pulsed ON-Off MH-induced release. Adapted with permission from reference 41, copyright 2020 John Wiley and Sons.

Recently, de Moura *et al.* presented the design, preparation, and proof-of-principle validation of novel magnetic hybrid nanowax composites, mWNVs, as temperature responsive MRI and chemotherapy/MH theranostic agents (Figure 14A) 41. These *Carnauba* wax spherical nanoparticles incorporated 8 nm magnetic nanoparticles and chemotherapy drug Oncocalixone A (Onco A) and were synthesised by a simple and scalable modified melt emulsification protocol and loaded with 0-7% iron oxide nanoparticles in respect to wax (w/w). These platforms showed very promising results for *T_2_*-weighted MRI, since the *r_2_* for compound S7 (7% MNPs) was 930 mM^-1^ s^-1^ at 1.4 T, which is much higher than the relaxivity of commercially available Endorem at that magnetic field strength (61 mM^-1^ s^-1^). The efficiency of mWNVs as MH effectors was also evaluated. All the magnetite-loaded samples showed high specific absorption rates (SAR) values >220 W g^-1^ Fe, therefore S7 was chosen for drug loading because it showed the largest *r_2_* of all the tested compounds. After Onco A loading into the wax nanocomposites, the drug release mechanism was studied using fluorescence measurements. Results showed that the chemotherapeutic drug release was enhanced in S7-Onco A nanocomposites in response to the temperature increase resulting from the application of a 24 mT (523 kHz) alternating magnetic field (Figure 14C and D). *In vitro* studies indicated a synergistic effect between thermo and chemotherapy, which is expected, since the drug release is a diffusive process that depends on heat generation and MH has been known to sensitise tumour cells to chemotherapy, in addition to direct thermal ablation. This proof-of-concept work takes a step forward in the design of highly controllable organic/inorganic lipid-based nanotheranostics and it is relevant for the design of future externally triggered drug release systems with integrated imaging.

### Ultrasound responsive smart theranostics

Ultrasounds are high frequency sounds waves with adjustable intensities for different applications, including imaging (< 20 kHz) or disruption of nanocarriers to trigger the release of encapsulated drugs or MRI CAs (> 20 kHz) 33.

Ultrasounds can also be used for therapy; high intensity focused ultrasound (HIFU) is a non-invasive therapeutic modality that focuses ultrasound waves to induce thermal ablation in tumours 165. HIFU has also been used to trigger the temperature-dependent generation of microbubbles in PFH/DOX@PLGA/Fe_3_O_4_-FA nanocomposites and thus serve as a MRI/US-guided HIFU/chemo synergistic therapy 166. These nanotheranostic agents were prepared by a double emulsion method and had DOX encapsulated in a temperature-responsive perfluorohexane (PFH) core and Fe_3_O_4_ nanoparticles in a PLGA shell. Folic acid was then added to the outer surface of the nanocomposites *via* carbodiimide reaction. *In vivo* studies were performed in hepatocellular tumour-bearing mice to evaluate the efficiency of these platforms as MRI CAs, US-imaging enhancers, HIFU sensitisers, chemotherapy agents and hyperthermia effectors. *In vitro* studies showed that exposure to HIFU generated a thermal effect that triggered PFH vaporisation of the NPs with around 49% efficiency. This translated into enhanced *in vivo* US imaging, as the echo enhancement and grey volume increased significantly after exposure to HIFU (5.91 to 128.1). The targeted nanocomposites could also be used for MRI, since they actively accumulated at tumour sites, with the highest contrast enhancement reached at 6 h post injection. The *in vivo* synergistic effect of the PFH/DOX@PLGA/Fe_3_O_4_-FA nanocomposites for HIFU/chemotherapy was then assessed. Treatment with the nanoplatforms led to significant tumour ablation, in comparison to untargeted nanocomposites, or injection of nanoparticles without HIFU exposure. This indicates that these theranostic agents responded to a HIFU stimulus and thus induced the release and increased the susceptibility towards anticancer drug DOX. PFH/DOX@PLGA/Fe_3_O_4_-FA nanocomposites are then examples of smart theranostics with remotely activated US imaging and HIFU/chemotherapy.

Rizzitelli *et al.* developed liposomes that released DOX and MRI CA Gadoteridol in response to pulsed low intensity non-focused ultrasounds (pLINFU) 167, 168. pLINFU are acoustic waves with intensity lower than 10 W cm^-2^ and US frequencies lower or within the therapeutic range (20 kHz-3 MHz) that allow for the release of nanocarriers' cargo through mechanical interactions, in a process that does not induce a temperature shift. These Gado-Doxo-Lipo liposomes were prepared by hydration of a thin lipid film and yielded nanoparticles with Gadoteridol and DOX encapsulated in a ratio of 11:1, which could help increase the local *T_1_* signal 167. *In vivo* studies on a syngeneic mouse model of breast cancer showed that the release and accumulation of DOX and Gadoteridol in tumours was triggered by a single treatment with pLINFU (3 MHz, total insonation time 2 min), as the generated waves induced the permeabilization of the tumour's vascular endothelium. Since Gadoteridol was released simultaneously to DOX, MRI was used to both confirm drug delivery to tumours and to assess the efficacy of the chemotherapy. These researchers then tested these liposomes in a mammary carcinoma model in BALB/C mice using a modified method where two sequential pLINFU shots were applied: the first to release the cargo from liposomes circulating in tumour blood vessels, and the second to increase tumour vessel permeability and thus facilitate the extravasation of the released cargo to tumour cells. This method once again allowed for the evaluation of the cargo release efficiency, as this process was accompanied by an MRI *T_1_* contrast enhancement in tumours and led to complete regression of the tumours. These liposomes are examples of US-responsive theranostics for MRI with remotely activated therapy.

### Light responsive smart theranostics

Using light as a stimulus has various advantages, including minimal invasiveness and the possibility to easily tune to a desired wavelength and induce a response in a specific system 169, 170. Light absorption by one or several moieties of the theranostic agents could induce photochemical reactions in those components to trigger drug release, and/or potentiate the interaction of photosensitisers with oxygen to generate singlet oxygen 171. Absorption of light by these molecules can also be used to facilitate PA or FL imaging, or to improve the therapeutic outcome, through PDT or PTT. Several light-sensitive theranostic agents have been proposed 36-40, 172.

Kim *et al*. used a light source to enhance the generation of singlet oxygen by photosensitiser Ce6 172. To do this, they developed Ce6-loaded manganese ferrite nanoparticle-anchored mesoporous silica nanoparticles (MFMSN-Ce6s) that function as theranostic agents for MRI-guided PDT. The nanoparticles work as Fenton catalysts that continuously generate oxygen under hypoxic conditions, which improves the ROS generation of photosensitisers in response to a 680 nm laser 173, thus enhancing the therapeutic effects of PDT. In terms of synthesis, 6 nm manganese ferrite nanoparticles were conjugated to 50 nm mesoporous silica nanoparticles by a nucleophilic substitution reaction, which was followed by functionalisation with PEG.

**Figure 15:**
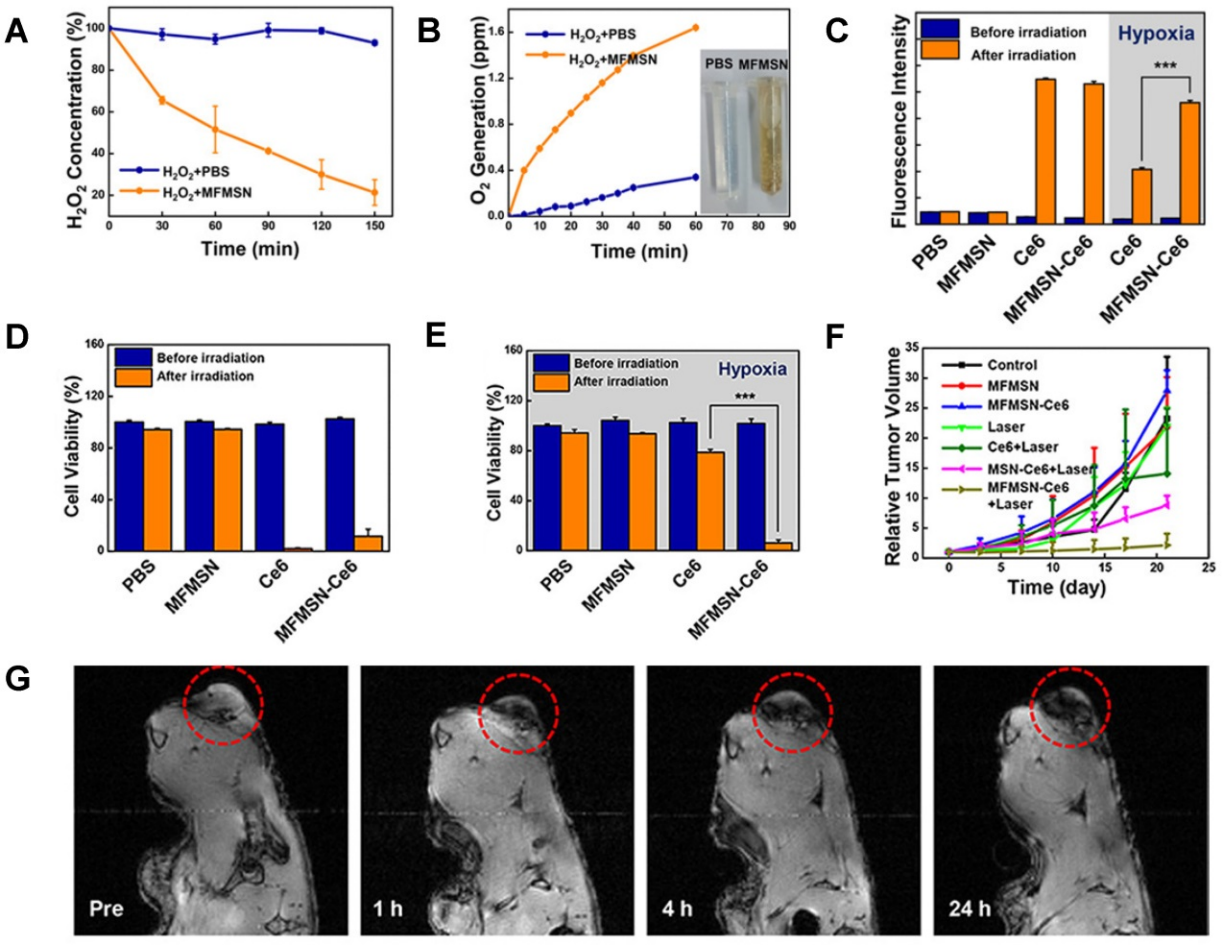
** A.** Degradation of H_2_O_2_ (n = 3) and **B** O_2_ generation after treatment with MFMSNs in PBS. **C.** Generation of singlet oxygen measured by the fluorescence intensity of SOSG (n = 3). **D.** Cell viability assay of MFMSN-treated U-87MG cells in normoxic environments. **E.** Cell viability assay of MFMSN-treated U-87MG cells in hypoxic environments. **F.** Tumour volume changes after treatment of U-87MG tumour-bearing mice. **G.** In vivo T_2_*-weighted MR images of a tumour-bearing mouse at various time periods, at 9.4 T. Tumours are circled with red dashed lines. Adapted with permission from 172, copyright 2017 American Chemical Society.

These nanoparticles had a hydrodynamic size below 100 nm and an *r_2_*= 60.9 mM^-1^ s^-1^ at 3 T. Ce6 was then loaded on the porous surface of the nanoplatforms. The Fenton catalytic efficiency of MFMSN-Ce6 was evaluated in time-dependent assays that measured the degradation of H_2_O_2_ and the generation of O_2_ under hypoxia conditions (Figure 15A and B). These nanoparticles were able to degrade the former molecule and generate the latter. The ability of Ce6 and MFMSN-Ce6 nanoconstructs to generate singlet oxygen species in response to a 680 nm laser (5 min) under normoxic and hypoxic conditions was also evaluated (Figure 15C). In non-hypoxia conditions, Ce6 and MFMSN-Ce6s were able to produce similar amounts of singlet oxygen. However, under hypoxia conditions, the nanoparticles produced 2.2 times more singlet oxygen in response to the laser. This effect translated into a higher therapeutic efficiency in hypoxia conditions during *in vitro* and *in vivo* studies. MTS assays showed that treatment with Ce6 and MFMSN-Ce6 had a similarly high PDT efficacy in U-87MG cells after laser irradiation under normoxic conditions (Figure 15D). However, in hypoxic conditions, the cell viability only decreases significantly in MFMSN-Ce6-treated cells (Figure 15E). *In vivo* studies showed a significant decrease in tumour volume after irradiation of tumour sites with a 670 nm laser (0.88 W, 5 min, 24 h post treatment) following injection of U-87MG tumour-bearing mice with MFMSN-Ce6 nanoparticles (Figure 15F), due to a higher accumulation of the theranostic agent at the tumour site and enhanced PDT effect under hypoxia conditions. The accumulation of MFMSNs in the tumour region was confirmed by *T_2_*-weighted MRI, which demonstrated higher signal attenuation at tumour site (Figure 15G).

CuS-MnS_2_ nanoflowers were designed as light-enhanced MRI-guided PTT/PDT agents 40. These nanoparticles showed good photothermal, photostability and photodynamic properties and had a light-to-heat efficiency of 67.5%. This was the first report of Cu-Mn-S nanomaterials boasting NIR light-responsive ROS generation, which accounted for their potent light-dependent anticancer efficacy in the treatment of A2780 tumour-bearing mice. The same principle was applied later for other Mn-based MRI-guided PTT theranostic agents 37.

**Figure 16:**
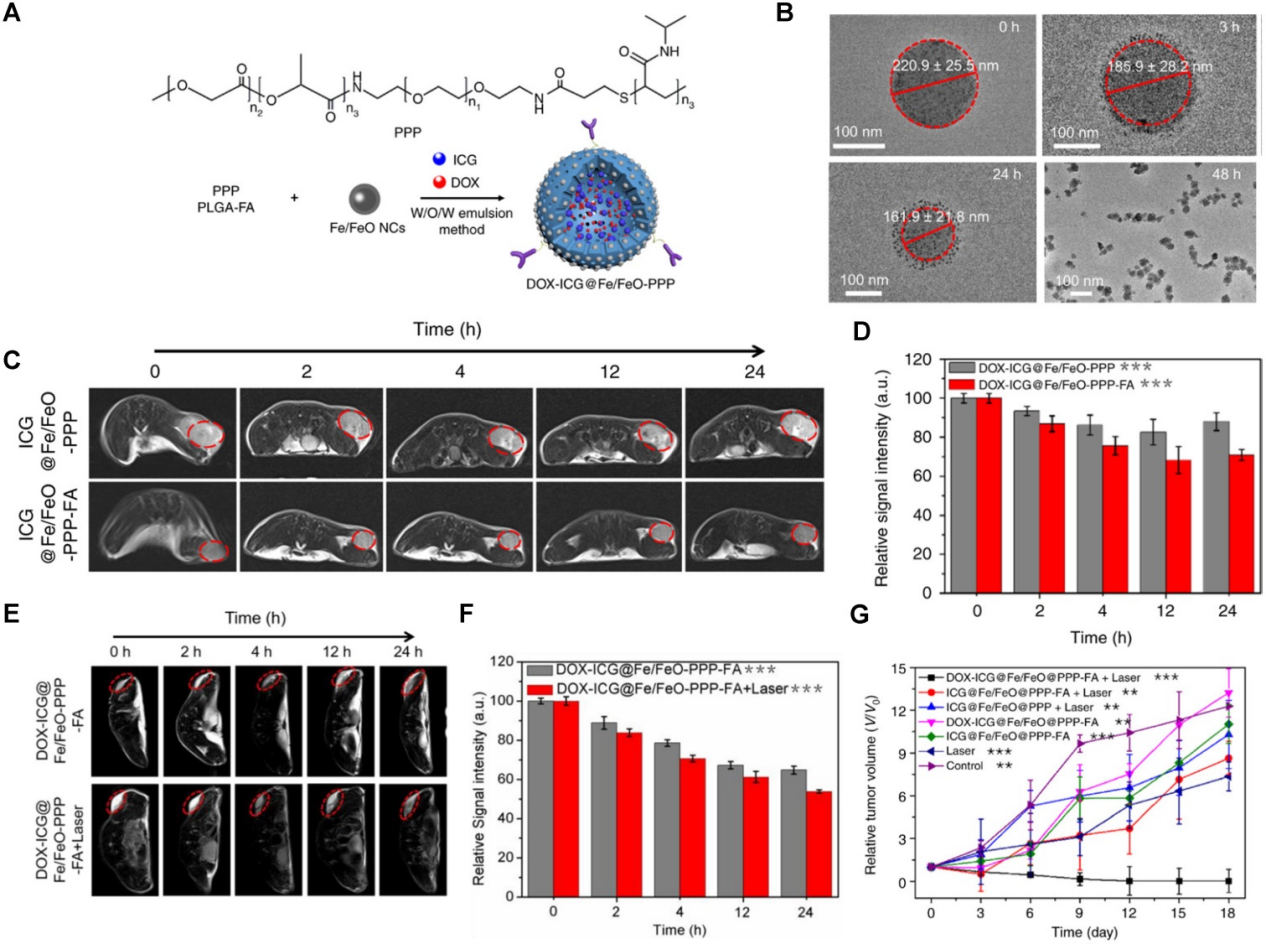
** A.** Fabrication of DOX-ICG@Fe/FeO-PPP nanocapsules. **B.** TEM image of the shrinking process for DOX-ICG@Fe/FeO-PPP nanocapsules 48 h after the irradiation of laser (808 nm, 0.3W cm^-2^) for 5 min (pH = 6.5). **C.** Real-time MRI of KB tumour-bearing mice after intravenous injection of ICG@Fe/FeO-PPP nanocapsules and ICG@Fe/FeO-PPP-FA nanocapsules at 3 T. **D.** The relative MRI signal intensities changing at the tumour site after intravenous injection of ICG@Fe/FeO-PPP nanocapsules and ICG@Fe/FeO-PPP-FA nanocapsules, respectively. **E.** Real-time MRI of KB tumour-bearing mice after intravenous injection of DOX-ICG@Fe/FeO-PPP-FA nanocapsules without and with the irradiation of laser (808 nm, 0.3 W cm^-2^, 5 min) respectively, at 3 T. **F.** The relative MRI signal intensities changing at the tumour site. **G.** Volume change of tumour in the different treatments (five mice per group). Adapted with permission from reference 106, copyright 2019 Springer Nature.

Wang *et al.* synthesised folate receptor targeted MRI theranostic nanocapsules, DOX-ICG@Fe/FeO-PPP-FA, for NIR light and tumour microenvironment-responsive PDT and chemotherapy 106. Interestingly, exposure of these particles to NIR light and acidic pH (pH = 6.5) induce drug release through a novel size-switchable mechanism. This is an example of how light absorption can trigger physical changes that allow for the release of encapsulated components from the theranostic agent. First, by tethering PLGA-polyethylene glycol-poly(N-isopropyl acrylamide), or PPP, to the iron nanoparticles, Fe/FeO-PPP heterostructures were synthesised. Chemotherapy agent DOX and photosensitiser ICG were then co-loaded into the Fe/FeO-PPP structures by water-oil-water emulsion (Figure 16A).

These nanoparticles had large average sizes (219 ± 52 nm), which might contribute to a longer blood circulation half-life but could hinder their ability to extravasate to tumour tissues 174, 175. In this work, a size-switchable strategy was employed to not only increase the blood circulation time, typical for larger 100-200 nm nanoparticles, but also to improve penetration into deep tumour tissues, which is usually easier for small 4-20 nm particles. In this case, TEM images demonstrate that nanoparticles shrunk from 221 ± 26 nm to 162 ± 22 nm after 24 h and then to 55 ± 4 nm 48 h after laser irradiation at pH = 6.5 (Figure 16B). This shrinkage and decomposition of the nanocapsules into small-sized nano-drugs is thought to be caused by loss of stability inside the nanoparticles, due to changes in interaction between the internal interface of the nanocapsules and water molecules by intermolecular forces of PPP and the degradation of ester and amide bonds in PPP at low pH. While the large nanoparticles remained in blood for longer, the small-nanodrugs could enter tumours and produce ROS by Fe^2+^-catalysed Fenton reaction while delivering DOX and ICG to cancer cells. The accumulation of nanocapsules at the tumour site was evaluated *in vivo* with *T_2_*-weighted MRI of KB tumour-bearing mice in the presence and absence of a laser (Figure 16C-F). Results show that ICG@Fe/FeO-PPP-FA nanocapsules enhance the *T_2_* signal intensity and make the tumour region darker than the non-targeted nanocapsules, after 24 h of injection. The contrast enhancement at the tumour site was even more pronounced after treatment with a laser, which suggested that the laser-trigged shrinkage of the DOX-ICG@Fe/FeO-PPP nanocapsules either induced deep tissue penetration of the theranostic agent or changed the relaxometric properties of the nanoparticles. The *in vivo* efficacy of these agents for cancer therapy revealed that injection of the nanocapsules followed by laser irradiation (808 nm, 0.3 W cm^-2^) was sufficient to eradicate KB tumours in mice (Figure 16G).

Overall, these results showed that the size-switchable strategy had a positive effect on the combinatorial therapy outcome and could, in the future, be adapted to fit a larger range of other functions while maintaining a multi-triggered controllable smart design.

## Conclusion and Future Perspectives

Cancer theranostics has emerged as a very promising multi-disciplinary field that is responsible for narrowing the divide between biomedical sciences, medical imaging and nanotechnology. Although theranostics is still a new field, and relatively unexplored, it is already paving the way for the advancement of personalised medicine and thus, is indirectly contributing to the improvement of the clinical care quality.

Smart cancer theranostics go a step further by providing a more controlled and more informative method to deliver drugs specifically to cancer cells by exploring biochemical shifts between healthy and pathological tissues. Additionally, MRI-assisted responsive theranostics make use of the best parts of MRI, such as minimal invasiveness, high tissue penetration, high spatial resolution and ability to better detect soft tissue pathologies, and, when necessary, can be combined with other imaging modalities to overcome the technique's known sensitivity limitations. The combination with other imaging modalities is made easier by the impressive repertoire of strategies that have been employed in the preparation of responsive and/or dual modal contrast agents for MRI.

In this review, different design strategies employed to prepare smart theranostic agents were presented, including small-molecule and nanoparticle-based approaches (Table 1). The latter are by far the more common strategy in the theranostics field, for several reasons. First, in terms of synthesis, it is easier to equip these nanosystems with multiple functions, whether targeting agents and/or more than one imaging or therapeutic modality, than small-molecule agents. Nanoparticle-based platforms can also be more controllable in terms of blood circulation half-life and target specific accumulation. Still, the mechanism of drug release and MRI signal switch of small molecule theranostics can be more straightforward than their nanosized counterparts. Although several nanoparticle systems have been reported in this review, the most extensively studied and effective in delivering therapeutic efficacy and higher contrast enhancement appear to be polymeric nanoparticles 32, 35, 105, 106, 113, 116, 117, 148, 172. This is likely related to the possibility of better controlling the drug and/or CA load during the adsorption, entrapment or encapsulation processes. An interesting subset of this smart theranostics repertoire includes externally responsive nanocarriers that transport small molecules into tumour cells, like the work of Wang *et al* 106. This kind of work can pave way for more controllable, perhaps multi-stimuli responsive systems that can release drugs or even other small-molecule theranostics safely to target sites, before activating those functions *in situ*.

Of the several responsive strategies of smart theranostics that have been presented here, only a few exhibit trigger-switchable MRI contrast enhancement. Theranostics featuring imaging agents that switch “on” or “off” after reaching the target of interest can provide valuable molecular information, that is not attainable with constructs bearing smart therapeutic effectors alone. The most successful theranostics featuring responsive contrast enhancement reported to date have been ESION or Mn^2+^-based nanosystems in which an internal stimuli triggers either an on/ON or OFF/ON switch of the MRI contrast enhancement 105, 113, 148. The higher efficacy of these turn “on” switches, when compared to turn “off” systems, in which the relaxivity decreases in response to the biological event, is reflected in a boosting of contrast enhancement upon reaching the target tissue, which can, in the case of theranostics, greatly facilitate drug response evaluation. In theranostics with OFF/ON, the drug release evaluation is especially accurate, since the *in vivo* signal enhancement is likely caused almost entirely in response to the stimulus and not due to a higher accumulation of the contrast agents in the tumours (as could happen in on/ON switches). Although this review shows some successful examples of theranostics with environmentally switchable CAs, future attempts at designing smart theranostic platforms should make use of the wide range of previously reported responsive MRI CAs to prepare smarter theranostic agents. Focusing specifically on the design of theranostics with an OFF/ON switch might also improve the accuracy of the drug release evaluation and thus better estimate the therapeutic outcome. Indeed, innovation in the design of MR theranostics inspired by some of the previously reported remotely activated MR CAs 52, 176 might yield theranostic systems equipped with switchable contrast enhancement induced by external triggers.

Regarding therapy, a large majority of the reported theranostics employ chemotherapy drugs in their designs, which is expected, since chemotherapy is the second most common cancer treatment in the UK, according to Cancer Research UK, following surgery. In these designs, the chemotherapy drug is usually released in response to a tumour microenvironment trigger. However, in some theranostics, the chemotherapy drug is released in response to an external trigger 35, 41. In this case, chemotherapy is used in combination with either PDT, PTT or MH. The combination of different types of therapies has a synergetic effect in several theranostic systems 35, 41, 106, 118. As such, future designs should attempt to combine at least two distinct types of activatable cancer therapy that are responsive to environmental and/or external triggers.

Overall, smart theranostic systems tend to be more therapeutically efficient and more diagnostically informative when they combine responsive therapy and switchable imaging simultaneously. Having multiple trigger responsive systems might also be helpful, in particular for therapy, as the combination of chemotherapy with a temperature-increasing therapeutic modality, such as PTT or MH, can make cancer cells more susceptible to drugs and thus improve the treatment outcome. Additionally, smart theranostic systems should strive to include switchable CAs in their design, as they can provide valuable information on cancer progression and treatment response.

Regarding the targeting strategies, Table 1 shows that passive targeting through the EPR effect is the most common amongst the provided examples. Targeting tumour tissues *via* EPR has proven successful for a variety of nanomedicines and nanotheranostics 177. Nonetheless, since the EPR effect depends on the tumours' leaky vasculature resulting from rapid angiogenesis and poorly developed lymphatic drainage system, it is highly conditioned by individual heterogeneity and by the stage of the malignancy 177-180. The blood circulation time of the theranostic agents can also impact tumour uptake. These factors will undoubtedly influence the accumulation of theranostic agents in target tissues and thus greatly impact the imaging and therapeutic efficiency of the constructs. Smart theranostics with active targeting components, such as ligands, might allow for early malignancy detection. Still, since most biochemical targets exist in the body at very low concentrations (e.g. receptors exist in membranes in concentrations ranging from 10^-9^ to 10^-3^ mol g^-1^ of tissue) 181, the imaging and therapeutic efficiency of these theranostics can be hindered unless a strategy is applied to ensure the minimal theranostic concentration threshold for therapeutic and imaging purposes is reached. This can be achieved by increasing the theranostic payload by using ratiometric approaches to conjugate multiple theranostic agents to a single targeting vector in order to increase the local concentration of a theranostic, or by making use of endogenous biological processes, such as passive transport, receptor-mediated internalisation, or endocytosis, to promote the intracellular accumulation of these agents.

While the future is bright and some of these responsive theranostic probes could be very promising in the growing era of personalised medicine, some hardships still need to be surmounted before clinical application. The discrepancy between the dose of contrast agent that needs to be administered to enhance MRI signal and the concentration of biological target or endogenous stimuli is one of the major obstacles. Not only does the solution require intelligent design of theranostics to ensure sufficient local contrast enhancement, but it also demands the agent to be manufactured in bulk and pass rigorous preclinical safety studies. The latter would obviously require a major investment, which might be difficult to obtain for tailored theranostic agents that would probably only be effective in a subset of patients. There are other barriers preventing the clinical application of responsive MRI theranostics, including concerns about their reproducibility. Environmentally responsive theranostics respond to internal triggers, which might be heterogenous between patients and might greatly vary in concentration, thus possibly hindering the reproducibility of these constructs. Remotely activated theranostics might, in theory, provide more reproducible outcomes, unless the pharmacokinetics and biodistribution of the theranostics varies considerably between individuals. Still, these responsive MR theranostic platforms could help with the treatment of a wide range of pathologies that stretch a lot further beyond cancer. Even though breakthroughs in this field are highly dependent on the cooperation between different areas of knowledge, the future of smart theranostics is promising.

## Figures and Tables

**Table 1:** Summary table of the smart theranostics discussed in this section and their pH-responsive mechanisms

Smart theranostic	Structure of responsive linker	Responsive trigger	Responsive Mechanism	Ref
PMN/Ce6	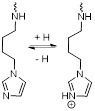	pH	**pH = 6.5-7**: Ionisation of imidazole groups cause swelling. **pH = 5.5**: Further ionisation of imidazole groups causes disassembly and release of photosensitiser.	113
DOX@ES-MION3@RGD2@mPEG3	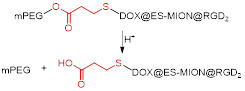	pH	**pH = 5.5**: Cleavage of β-propionate linker leads to exposure of RGD2 ligand and tumour cell internalisation.	119
Gd_2_O_3_@MSN-DOX	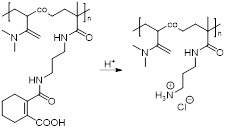	pH	**pH = 5.0**: Charge reversal and disintegration of polyelectrolyte P(DMA-co-TPAMA) leads to DOX release.	115
MTX-Mn@PEG	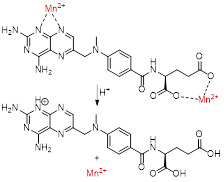	pH	**pH = 5.5**: Protonation of carboxylic acids and nitrogen heterocycle of MTX reduces coordination and releases Mn^2+^ from MTX complex.	117
SPA-SPIO-IR780	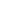 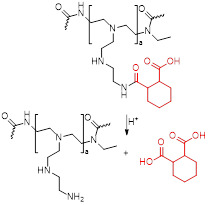	pH	**pH = 6.5**: Cleavage of bond between PEI and HHPA and exposure of positive amino groups enhances tumour cell internalisation.	116
Fe_3_O_4_@TA-PEG/ICG	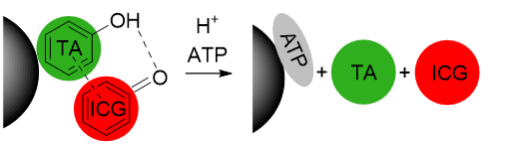	pH ATP	**pH = 5.5**: Protonation of hydroxyl groups of TA leads to disassembly by weakening of coordinate and hydrogen bonds. Quenching of ICG is reduced. ATP (10 mM): Displacement of TA from the nanoparticles and disassembly.	32
Bi-MSx-PEG/DOX NFs	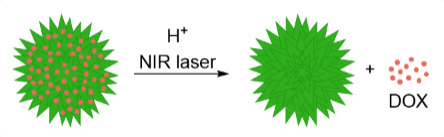	pH NIR light	**pH = 5.5**: Solubility of DOX is increased. NIR laser: Temperature increase reduces binding of DOX to NPs, facilitating its release.	118
MCDION-Se	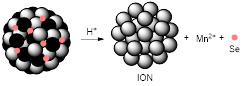	pH	**pH = 5.5**: Degradation of MnCO_3_ component of nanoparticles leads to release of Mn^2+^ and Se in tumour cells.	128
Gd-texaphyrin-DOX complex	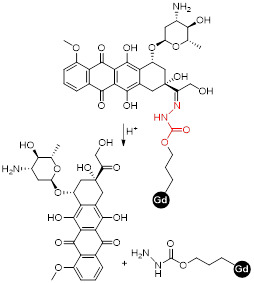	pH	**pH = 5.5**: Cleavage of hydrazone linker leads increase of FL signal of DOX due to separation from the Gd-texaphyrin complex.	132

**Table 2:** Summary table of the smart theranostics discussed in this section and their redox-responsive mechanisms

Smart theranostic	Structure of responsive linker	Responsive trigger	Responsive Mechanism	Ref
Gd-DOTA-Biotin-CPT complex	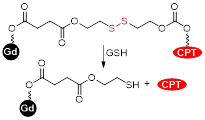	Redox	GSH (5 mM): Cleavage of disulphide linker causes release of CPT and an increase in FL signal intensity.	140
HSPt-PEG-SPION	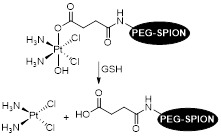	Redox	GSH (2 mM): Reduction of Pt(IV) complex to a Pt(II) species causes dissociation of HSPt-PEG-SPIONs and release of active drug cisplatin.	147
DOX/MnO_2_@PVCL NGs	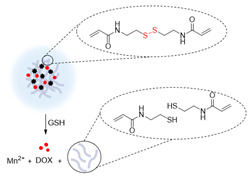	Redox	GSH (10 mM): Cleavage of disulphide bonds within nanogel polymers and decomposition of MnO_2_ nanoparticles to Mn^2+^ ions lead to the release of DOX and increase of the MR signal.	148
Magnetic AuNWs	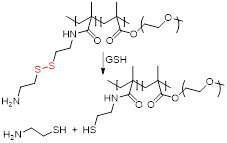	Redox NIR light	GSH (10 mM): Cleavage of disulphide bonds in polymers induces disassembly of AuNWs and release of ESIONs.	105
NP-RGD	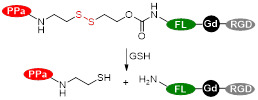	Redox NIR light	GSH (10 mM): Cleavage of disulphide and disassembly of NP-RGD nanoparticles.	153
MS@MnO_2_-CPT NPs	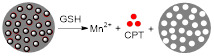	Redox	GSH (10 mM): Decomposition of MnO_2_ nanoparticles to Mn^2+^ ions and subsequent release of previously blocked CPT from MS NPs.	154

**Table 3:** Summary table of the smart theranostics discussed in this section and their enzyme-responsive mechanisms

Smart theranostic	Structure of responsive linker	Responsive trigger	Responsive Mechanism	Ref
ATF-IONP-Gem	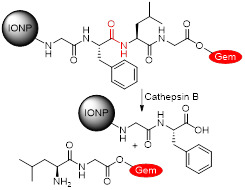	Enzyme (cathepsin B)	Cathepsin B (1 µM): Cleavage of GFLG tetrapeptide linker induces release of anticancer drug Gem from system.	157
pHPMA-Gd-PTX-Cy5.5	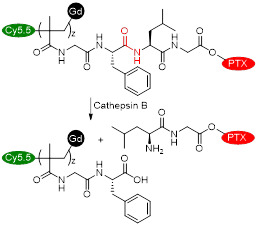	Enzyme (cathepsin B)	Cathepsin B (2.8 µM): Cleavage of GFLG tetrapeptide linker induces release of anticancer drug PTX from system.	158
BP-PTX Gd NPs	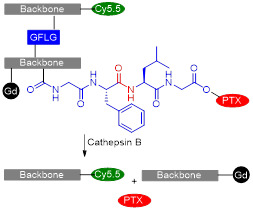	Enzyme (cathepsin B)	Cathepsin B (2.8 µM): Cleavage of GFLG linkers connecting branched pHPMA polymers and binding PTX to the system induces nanoparticle disassembly and release of anticancer drug PTX.	28
T-MAN	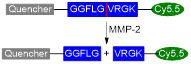	Enzyme (MMP-2)	MMP-2 (10 nM): Cleavage of GGFLGVRGK peptide results in the release of quencher QSY21 from T-MAN and increase of the fluorescent signal intensity of Cy5.5.	162

**Table 4:** Summary table of the smart theranostics discussed in this review and their imaging and therapeutic modalities, responsive triggers, targeting strategies and MRI properties

Smart theranostic	Classification	Imaging Modality	Therapy	Responsive trigger	Targeting strategy	Type of MRI CAs	*r_1_* or *r_2_* (mM^-1^ s^-1^)	Ref
PMN/Ce6	Environmentally switchable CAs; Remotely activated therapy.	MRI FL	PDT	pH	EPR effect	*T_1_* (ESIONs)	*r_1_* = 3.307 (pH = 7.4, 1.5 T) *r_1_* = 3.87 (pH = 5.5, 1.5 T)	113
DOX@ES-MION3@RGD_2_@mPEG3	Environmentally activated therapy	MRI	Chemo	pH	Ligand targeting	*T_1_* (ESIONs)	*r_1_* = 2.34 (7 T)	119
Gd_2_O_3_@MSN-DOX	Environmentally switchable CAs; Environmentally activated therapy.	MRI	Chemo	pH	Ligand targeting	*T_1_* (Gd^3+^)	*r_1_* = 9.14 (3 T)	115
MTX-Mn@PEG	Environmentally activated therapy	MRI	Chemo	pH	EPR effect	*T_1_* (Mn^2+^)	*r_1_*= 7.76 (0.5 T)	117
SPA-SPIO-IR780	Remotely activated therapy	MRI NIFI	PDT	pH	EPR effect	*T_2_* (SPIONs)	*r_2_* = 254.09 (1.5 T)	116
Fe_3_O_4_@TA-PEG/ICG	Environmentally switchable CAs; Remotely activated therapy.	MRI FL	PTT	pH ATP	EPR effect	*T_2_* (SPIONs)	*r_2_*= 251.2 (pH = 7.4, 0.5 T) *r_2_*= 13.68 (pH = 5.5, 10 mM ATP, 0.5 T)	32
Bi-MSx-PEG/DOX NFs	Environmentally activated therapy; Remotely activated therapy.	MRI CT PA	PTT; PDT; RT; Chemo (DOX)	pH NIR light X-rays	EPR effect	*T_1_* (Mn^2+^)	*r_1_* = 2.60 (0.5 T)	118
MCDION-Se	Environmentally switchable CAs; Environmentally activated therapy.	MRI	CDT	pH	EPR effect	*T_1_* (Mn^2+^)	*r_1_ =* 2.7 (pH = 7.4, 9.4 T) *r_1_ =* 7.1 (pH = 5.5, 9.4 T*)*	128
Gd-texaphyrin-DOX complex	Environmentally switchable imaging (FL); Environmentally activated therapy.	MRI FL	Chemo	pH	EPR effect	*T_1_* (Gd^3+^)	*r_1_* = 20.0 (1.4 T)	132
GdL complexes	Environmentally activated therapy	MRI	Chemo (Cisplatin)	Redox	EPR effect	*T_1_* (Gd^3+^)	*r_1_ =* 6.3 (0.5 T)	137
Gd-DOTA-Biotin-CPT complex	Environmentally switchable imaging (FL); Environmentally activated therapy.	MRI FL	Chemo (CPT)	Redox	Ligand targeting	*T_1_* (Gd^3+^)	*r_1_* = 8.3 (0.5 T)	140
HSPt-PEG-SPION	Environmentally activated therapy	MRI	Chemo (Cisplatin)	Redox	EPR effect	*T_2_* (SPIONs)	*r_2_*= 228.96 (0.5 T)	147
DOX/MnO_2_@PVCL NGs	Environmentally switchable CAs; Environmentally activated therapy	MRI	Chemo (DOX)	Redox	EPR effect UTMD	*T_1_* (Mn^2+^)	*r_1_* = 0.04 (no GSH, 0.5 T); *r1* = 8.33 (10 mM GSH)	148
Magnetic AuNWs	Environmentally switchable CAs; Remotely activated therapy	MRI PA	PTT	Redox NIR light	EPR effect	*T_1_* (ESIONs)	*r_1_*= 1.1; *r_2_*= 198.6 (no GSH, 7 T); *r_1_*= 3.2; *r_2_*= 33.9 (10 mM GSH)	105
NP-RGD	Environmentally switchable imaging (FL); Remotely activated therapy	MRI FL	PDT	Redox NIR light	Ligand targeting	*T_1_* (Gd^3+^)	*r_1_* = 20.0 (0.5 T)	153
MS@MnO_2_-CPT NPs	Environmentally switchable CAs; Environmentally activated therapy	MRI	CDT Chemo (CPT)	Redox	EPR effect	*T_1_* (Mn^2+^)	*r_1_*= 0.5 (no GSH); *r_1_*= 6.91 (1 mM GSH)	154
PAA-IONPs	Environmentally switchable imaging (FL); Environmentally activated therapy	MRI FL	Chemo (Taxol)	Enzyme (esterease) pH	Ligand targeting	*T_2_* (SPIONs)	*r_2_* = 202.0 (0.5 T)	160
ATF-IONP-Gem	Environmentally activated therapy	MRI	Chemo (Gem)	Enzyme (cathepsin B)	Ligand targeting	*T_2_* (SPIONs)	*r_2_*= 195 (3 T)	157
pHPMA-Gd-PTX-Cy5.5	Environmentally activated therapy	MRI FL	Chemo (PTX)	Enzyme (cathepsin B)	EPR effect	*T_1_* (Gd^3+^)	*r_1_* = 12.9 (1.5 T)	158
BP-PTX Gd NPs	Environmentally activated therapy	MRI FL	Chemo (PTX)	Enzyme (cathepsin B)	EPR effect	*T_1_* (Gd^3+^)	*r_1_*= 8.6 (1.5 T)	28
T-MAN	Remotely activated therapy; Environmentally activated imaging (FL)	MRI FL	PTT	Enzyme (MMP-2)	Ligand targeting	*T_1_* (Gd^3+^)	*r_1_* = 60.0 (1 T)	162
Mn^2+^-PDA @DOX/PLGA	Remotely activated therapy	MRI	Chemo (DOX) PTT	Temp. NIR light	EPR effect	*T_1_* (Mn^2+^)	*r_1_*= 30.49 (0.5 T)	35
mWNVs	Remotely activated therapy	MRI	Chemo (OncoA) MH	Temp. AMF	EPR effect	*T_2_* (SPIONs)	*r_2_* = 930 (1.4 T)	41
PFH/DOX@PLGA/Fe_3_O_4_-FA	Remotely activated therapy; Remotely activated imaging (US)	MRI US	Chemo (DOX) HIFU	HIFU	Ligand targeting	*T_2_* (SPIONs)	*-*	166
Gado-Doxo-Lipo	Remotely activated therapy	MRI	Chemo (DOX)	US	EPR	*T_1_* (Gd^3+^)	*-*	167, 168
MFMSN-Ce6	Remotely activated therapy	MRI	PDT	NIR light	EPR effect	*T_1_* (Mn^2+^)	*r_1_*= 60.9 (3 T)	172
CuS-MnS_2_	Remotely activated therapy	MRI	PTT PDT	NIR light	EPR effect	*T_1_* (Mn^2+^)	*r_1_*= 28.5 (3 T)	40
DOX-ICG@Fe/FeO-PPP-FA	Remotely activated therapy; Environmentally activated therapy	MRI	PDT; Chemo (DOX)	NIR light pH	Ligand targeting	*T_2_* (SPIONs)	*r_2_*= 130 (3 T)	106
